# aTRAM - automated target restricted assembly method: a fast method for assembling loci across divergent taxa from next-generation sequencing data

**DOI:** 10.1186/s12859-015-0515-2

**Published:** 2015-03-25

**Authors:** Julie M Allen, Daisie I Huang, Quentin C Cronk, Kevin P Johnson

**Affiliations:** Illinois Natural History Survey, University of Illinois, Champaign, IL 61820 USA; Department of Botany and Beaty Biodiversity Centre, University of British Columbia, Vancouver, BC V6T 1Z4 Canada

**Keywords:** Massively parallel sequence data, Next-generation sequencing, Targeted gene assembly, Short-read archive, Phylogenomics, Phylogenetics

## Abstract

**Background:**

Assembling genes from next-generation sequencing data is not only time consuming but computationally difficult, particularly for taxa without a closely related reference genome. Assembling even a draft genome using *de novo* approaches can take days, even on a powerful computer, and these assemblies typically require data from a variety of genomic libraries. Here we describe software that will alleviate these issues by rapidly assembling genes from distantly related taxa using a single library of paired-end reads: aTRAM, automated Target Restricted Assembly Method. The aTRAM pipeline uses a reference sequence, BLAST, and an iterative approach to target and locally assemble the genes of interest.

**Results:**

Our results demonstrate that aTRAM rapidly assembles genes across distantly related taxa. In comparative tests with a closely related taxon, aTRAM assembled the same sequence as reference-based and *de novo* approaches taking on average < 1 min per gene. As a test case with divergent sequences, we assembled >1,000 genes from six taxa ranging from 25 – 110 million years divergent from the reference taxon. The gene recovery was between 97 – 99% from each taxon.

**Conclusions:**

aTRAM can quickly assemble genes across distantly-related taxa, obviating the need for draft genome assembly of all taxa of interest. Because aTRAM uses a targeted approach, loci can be assembled in minutes depending on the size of the target. Our results suggest that this software will be useful in rapidly assembling genes for phylogenomic projects covering a wide taxonomic range, as well as other applications. The software is freely available http://www.github.com/juliema/aTRAM.

**Electronic supplementary material:**

The online version of this article (doi:10.1186/s12859-015-0515-2) contains supplementary material, which is available to authorized users.

## Background

Short read sequencing methods have rapidly increased the amount of genetic data that can be obtained in a cost effective manner [1]. The computational skills and time necessary to assemble genes from these short read datasets is quickly increasing. To assemble genomic datasets researchers must first create a genome assembly using either a *de novo* or reference-based approach, if a reference genome is available [2]. Complete *de novo* genomic assemblies typically require a variety of DNA sequencing libraries and the assemblies are computationally intensive. Although reference-based assemblies can significantly reduce the computational time needed and be performed from a single DNA sequencing library, such assemblies can be problematic or impossible for more divergent taxa [3]. For many studies however (e.g. phylogenetics, gene family analysis), researchers may not need a complete genome assembly; rather the analysis may only require homologous sequencing data that covers all of the taxa of interest, and not genomic assemblies. As more and more genomic data become available, the time associated with these assemblies will become more challenging particularly for projects with hundreds of taxa. Approaches that target specific loci or genes from short read datasets will likely reduce the time necessary to assemble genetic datasets.

A few target-based methods have been made available that are shown to work well for very closely related taxa [4,5], RAD-PE fragments [6] and meta-genomic datasets [7]. However, a method is still needed that can target and assemble genes across highly divergent taxonomic datasets. In this article we describe aTRAM, automated Target Restricted Assembly Method, a software package designed to rapidly assemble genes using a single paired-end genomic library from divergent taxa. The aTRAM software is inspired by the Target Restricted Assembly Method (TRAM) idea first outlined by Johnson et al. [8]. TRAM used a targeted approach with local BLAST [9] to assemble genes from short sequencing reads. The aTRAM software is TRAM completely redesigned and fully automated including a number of optimizations to speed up gene assembly, as well as providing computational pipelines for multiple taxon datasets and downstream processing.

The aTRAM software distributes queries of the reads by using a MapReduce approach to parallelize indexing and searching of the short-read dataset. To assemble genes, aTRAM uses a query sequence, searches the short read databases for matches to the gene of interest, finds the matching mates, and uses a *de novo* assembler to assemble those reads. The aTRAM software then uses those contigs as the query sequence in the next iteration and repeats the process to completely assemble the locus of interest. We compare the results from aTRAM to those assembled using reference-based and completely *de novo* assemblies. Finally, we demonstrate the ability of aTRAM to assemble genes from highly divergent taxa.

## Implementation

The aTRAM package is downloadable from GitHub, (www.github.com/juliema/aTRAM) and is written as a Perl package that links together widely-cited programs in a novel way. These programs include BLAST [9]; two alternate *de novo* assemblers, Velvet [10] and Trinity [11]; and two multiple sequence aligners, MUSCLE [12] and MAFFT [13], aTRAM was designed so that new assembly and alignment software can be added as they become available. aTRAM has two components. The first component constructs an aTRAM formatted BLAST database from the original paired-end FASTQ or FASTA file and is performed once per sample. The second component is the search for a locus of interest, using an iterative approach aTRAM queries all or a fraction of the constructed short read database for the locus of interest and performs a *de novo* assembly. The package also includes post-processing scripts for validation of the results and pipeline scripts that automate multiple gene alignments across a number of short-read datasets.

### Database creation

**Figure 1:**
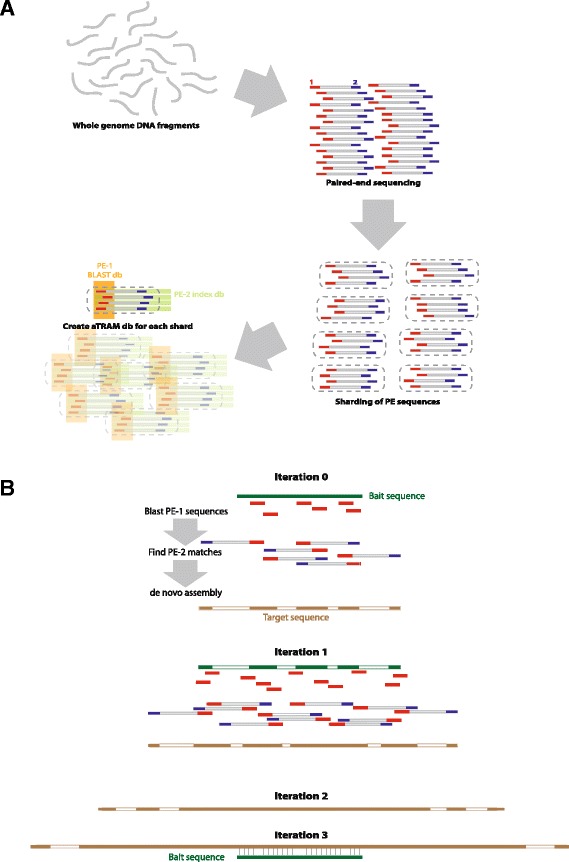
**Graphic of the aTRAM method. A)** Formation of the aTRAM database; DNA is sequenced into a paired-end short read dataset (SRD). aTRAM splits the SRD into shards, creates a BLAST formatted database of the first pair and indexes the paired-end for the sequences in each shard. **B)** In iteration 0 a query sequence in either amino acid or DNA format is queried against the aTRAM formatted database using BLAST. The top-hits and their paired-ends are selected and assembled *de novo*. In the following iterations the contigs from the previous iteration are queried against the same database using BLAST, the top-hits and paired-ends selected and assembled *de novo* until the full locus is assembled.

The aTRAM software creates a database from a paired-end FASTA or FASTQ sequence file using a MapReduce strategy [14]. Because sequence names are unrelated to the genomic content of the reads, the MapReduce strategy speeds up subsequent searches by a hashing function to distribute the reads across many partitions, or shards. The sizes of the shards are approximately equal and each should contain a random sample of reads from the original run. Searches can now be done more efficiently across a smaller subset of the whole dataset. For each shard, a BLAST database is constructed, corresponding to one end of each paired-end read in the shard. The mate of each read is placed in an easily searchable file (Figure 1A). This sharding process allows aTRAM to be parallelizable, because each shard can be searched independently on its own process. Furthermore, because each shard contains a random sample of the full short read dataset, any number of shards can be searched in an aTRAM run, allowing the user to vary the coverage depth used in the assembly and reducing computational time if genomic coverage is high.

### Gene assembly

The aTRAM pipeline iteratively searches the formatted database to assemble the gene of interest. Each shard is searched independently and the results are combined for *de novo* assembly. The target sequence is provided as either a DNA or amino acid FASTA file. For the first iteration, aTRAM uses BLAST to search each shard for reads that are similar to the target sequence. The top hits and their mates are retrieved, combined across all shards, and used as input to a *de novo* assembler (Velvet or Trinity). The possibility exists for other *de novo* assemblers to be written into aTRAM as plugins in the future [15-19]. The resulting contigs are then compared to the original target sequence using BLAST, and the most similar ones used as target sequences in the next iteration (Figure 1B). Because the subsequent iterations are using target sequences that were assembled directly from the short read database, further iterations will involve short reads that are not just similar but identical to the contig being assembled. The program stops when the total number of iterations determined by the user have been completed, or if the resulting contigs from any iteration matches exactly the contigs from the previous iteration. Alternatively, an *autocomplete* flag can be set to end the search if one of the contigs has sequence matching both the beginning and the end of the query sequence, suggesting that the contig includes the entire target. As mentioned, aTRAM can be adjusted to use a fraction of the available shards: the fraction should be calculated based on expected coverage of the target locus in the sequencing run, for example, a short read dataset that contains 5x coverage of the nuclear genome may contain 200x coverage of the ribosomal DNA and 800x coverage of the chloroplast genome [20]. Although 20-50x coverage may be optimal for many genes, it has been suggested that this method can work with coverage as low as 5x [8].

### Pipelines

The aTRAM package also includes ready-made pipelines for running aTRAM on multiple samples for many target sequences. *AssemblyPipeline* runs aTRAM on multiple target sequences for multiple samples; it is ideal for quickly producing a list of putatively orthologous genes from different species. *AlignmentPipeline* produces a set of aligned homologous sequences for a set of genes and a set of samples, allowing straightforward production of multiple gene alignments for gene tree analyses.

### Performance

#### aTRAM Compared to other methods

To compare the performance of aTRAM to genome assembly based methods and verify similar results a dataset of 1,534 single copy orthologs from *Pediculus schaeffi*, the chimpanzee louse, was chosen. These genes were first assembled using a reference-based approach against the body louse genome *P. humanus* in Johnson et al. [21]. Their study used one lane of an Illumina HiSeq2000 run, which resulted in 36 GB of data and over ~100X coverage of the genome (NCBI; SAMN02438447). The authors used CLC Genomics Workbench (CLCbio) to map paired-end reads to the reference genome and verified orthology using a reciprocal best-BLAST test (http://dx.doi.org/10.5061/dryad.9fk1s). The same set of genes was assembled using aTRAM and a completely *de novo* approach to compare the three sequence retrieval methods.

An aTRAM database was created from the same *P. schaeffi* paired-end library from Johnson et al. [21] taking a total of 2.37 hours to format the 36 GB library. The 1,534 reference *P. humanus* proteins were used as the target sequences for aTRAM assembly. Because the expected coverage of the genome for the complete Illumina run was over 100x, aTRAM was run using only 25% of the available shards, providing an estimated genomic coverage of ~25x. The program was set to run for five iterations using the *autocomplete* option. This run was performed on the Institute for Genomic Biology's Biocluster at the University of Illinois, which uses two Intel Xeon E5530 2.4GHz quad-core processors per node with 24 GB RAM per node. Both aTRAM steps were run on one node with four processors.

Finally, the same *P. schaeffi* paired-end library was used to create a completely *de novo* assembly. The raw reads were trimmed for nucleotide bias at the 5′ end and for low-quality bases at the 3′ end using the FASTX toolkit and error-corrected using Quake [22] with c = 2.83 for 19-mers. Paired-end reads were assembled in SOAPdenovo v1.05 [19] using K = 49, which is roughly half of the read lengths, and the optional GapCloser v1.10 algorithm with a minimum overlap = 31. Finally, the 1,534 genes were identified by creating a BLAST-formatted database of the *de novo* assembled contigs and using the *P. humanus* transcripts as targets for a BLAST search. The top hits were selected as the *de novo* contigs.

The aTRAM contigs, top hits from the *de novo* assembly, and the reference-based assembly sequences were each aligned against the original *Pediculus humanus* reference DNA sequences using MAFFT [13] with the included post-processing *PercentCoverage* script. Uncorrected p-distances (proportion of sites with differing nucleotides, not corrected with a model of molecular evolution) were calculated using a custom Perl script used originally in Johnson et al. [21] (Available on Github: juliema/publications/). Orthology was verified for the aTRAM and *de novo* contigs with the same reciprocal best-BLAST test that was previously used for the reference-based assemblies in Johnson et al. [21]. Because each method used BLAST for assembly the resultant contigs were then reciprocally compared to the entire *Pediculus humanus* protein coding genome, and if the original query sequence was the top hit, the assembled gene was considered to be orthologous to the query gene. Finally, the outputs from aTRAM, the reference-based assembly, and *de novo* assembly were aligned to each other and uncorrected p-distances calculated to determine if the three methods produced the same sequence for each gene.

#### aTRAM and Divergent Taxa

Samples of six species of lice were sequenced on an Illumina sequencer combining two species in a lane (NCBI: SAMN03360966 – SAMN03360971). Four species were sucking lice from the suborder Anoplura and thought to range from 25 – 75 million years divergent from the reference sequence *P. humanus* [23]. The other two species were chewing lice from the suborder Ischnocera and thought to be ~ 110 million years divergent from the reference species [24]. Johnson et al. [8] had previously identified a set of 1,107 genes as single copy orthologs protein coding genes across nine insect genomes, including lice, using OrthoDB [25]. The amino acid sequences from *P. humanus* for these 1,107 genes were used as query sequences in aTRAM for each of the six louse species. Each aTRAM contig was compared to the entire *P. humanus* protein-coding genome using the reciprocal best-BLAST test for orthology. The orthologous contigs were then aligned back to the *P. humanus* genome and uncorrected p-distances were calculated. To determine if a DNA query would also assemble genes across the divergent datasets, we ran 10 genes using the DNA from the reference *P. humanus*, and only those from the congener *Pediculus schaeffi* assembled. This suggests that aTRAM can be limited by the success of the initial BLAST search (Additional file 1), and as taxa become more divergent, amino acid sequences are the more optimal target sequence.

## Results and discussion

The aTRAM software rapidly assembles genes of interest from short paired-end sequencing reads, even across divergent taxa by iteratively querying and assembling reads. The MapReduce strategy [14] used in aTRAM enables faster searching of large short read data files, by splitting the short – read database into shards. Thus, the search is divided into many smaller parallelizable problems, speeding up computation time. This method also provides a means for further reducing computational time by allowing the user to search only a fraction of the short reads if genomic coverage is expected to be high.

### Comparisons with reference and de novo assemblies

Using aTRAM, we quickly assembled the sequences of 1,534 putatively single copy genes from the *Pediculus schaeffi* short read dataset. A total of 90% of the genes completely assembled before the fifth iteration, and 75% of those finished at the first iteration, taking a mean of 55 seconds per gene. Assemblies of the other genes ranged from 3-7.5 minutes. Although 170 genes were not flagged as complete by the fifth iteration of aTRAM, searching among the best contigs of these genes verified that many had the complete gene but were not flagged in the autocomplete process. These genes had a mean of 96.97% of the gene assembled, with a median of 99.46%. Further investigation revealed three typical reasons the genes were not marked as complete: 1) some were missing one section of the gene, 2) some had high sequence divergences as compared to the reference, and 3) others had a small exon at one end of the gene. Because the original query sequence only included exons and aTRAM assemblies include introns, genes with a small exon at one or both ends are unlikely to have a high BLAST match of these small exons back to the original gene sequence. These results suggest that even though the gene may not be flagged as complete by the end of the iterations, the entire gene may still be assembled. Furthermore, in our experience, as the assembled contig grows with each iteration adding more iterations allows the complete assembly of the locus of interest, this is particularly true for very large genes, where more iterations may be needed to completely assemble the gene.

### Gene completeness

When compared to the *P. humanus* reference sequence, aTRAM assembled a greater fraction of the gene than either the reference-based or *de novo* approaches (Table 1; Additional file 2). One possible explanation is that compared to the reference based assembly, aTRAM is more likely to assemble sequences at intron-exon junctions or at the 5′ and 3′ ends of genes.

**Table 1:** **Results from assembling 1,534 protein coding genes using aTRAM, a reference-based and a de novo approach**

	**Proportion (mean, range)**	**P-Distance**	**Reciprocal best-blast**
		**(mean, std dev)**	**(total 1,534)**
*aTRAM*	0.99 (1-0.20)	0.093 (0.044)	1,530 (99.7%)
*Reference*	0.93 (1-0.19)	0.077 (0.022)	N/A
*de novo*	0.92 (1-0.16)	0.095 (0.052)	1,512 (98.92%)

### Genetic distance to the reference

**Figure 2:**
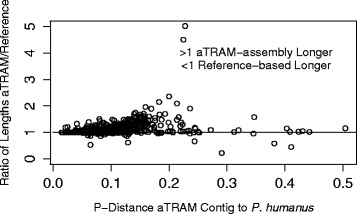
**Y axis is the ratio of the length of the contig assembled with aTRAM by the length of the contig assembled with the reference based approach.** Points under the 1 line are longer with the reference based approach and those above the line are longer from aTRAM assemblies. The x-axis indicates the uncorrected p-distance comparing the aTRAM contigs to the reference DNA sequence. The graph illustrates that aTRAM assemblies tended to be longer and the longer genes tended to be the more divergent ones, suggesting that aTRAM can assemble more divergent sections than a reference based approach.

The contigs returned from all three methods were similarly divergent when compared to their *P. humanus* orthologs, with the aTRAM and the *de novo* contigs having a few genes with higher distances. The mean p-distance to *P. humanus* was lowest for the reference-based contigs, most likely because more divergent regions failed to assemble (Figure 2). The aTRAM contigs had the next lowest p-distance, followed by the *de novo* contigs. All but four of the aTRAM contigs passed the reciprocal best-BLAST test of orthology, whereas 22 of the *de novo* contigs did not pass the test (Table 1). All of the reference-based contigs had previously passed the reciprocal best-BLAST test in Johnson et al. [21] and this resulted in the selection of the gene set used in our current comparisons.

### Sequence similarity among methods

Finally, the sequences from all three methods were compared to each other to determine if they assembled the same sequence. The contigs from each method were identical in many cases; when they were not identical, aTRAM contigs tended to be more similar to the reference-based contigs (mean uncorrected p-distance = 0.011) than to the *de novo* contigs (mean = 0.022). The *de novo* contigs tended to be less similar to either of the other methods overall, suggesting that the *de novo* contigs were the least accurate of the three methods tested. This may be a function of the *de novo* assembly method and other assemblers may perform better. Additionally, we aligned aTRAM contigs to previously Sanger-sequenced loci and found identical sequences for two of the three genes, the third gene was only different for two base pairs out of 241 bp and a single N in the Sanger sequence (Additional file 3). Taken together, these results suggest that the contigs assembled by aTRAM are of a similar (or higher) length and quality to those assembled using alternate methods, while taking a fraction of the time to assemble. The alignments from these methods have been made available from the Dryad Digital Repository http://dx.doi.org/10.5061.dryad.kh886.

### Assembling genes from divergent taxa

Finally, we used aTRAM to assemble genes from highly divergent taxa from *P. humanus*. Specifically, we assembled 1,107 1:1 orthologous genes from lice ranging from 25–110 million years divergent from the reference sequence [23,24]. aTRAM assembled nearly all of the genes from each of the six divergent taxa, ranging from 97% to 99% recovery (Table 2; Dryad Digital Repository http://dx.doi.org/10.5061.dryad.kh886). It is possible that some of genes that did not assemble were not present in the genomes of those taxa, having been lost over time. Between 2% and 6% of the assembled contigs did not pass the reciprocal best-BLAST test of orthology, leaving well over 1,000 genes for each species that did pass, suggesting these genes are orthologous to the reference gene and can be used for phylogenomic datasets. The mean p-distance from *P. humanus* for these genes ranged from 0.24–0.30. As expected, the more distantly related lice had higher p-distances from the reference sequences.

**Table 2:** **Results from assembling 1,107 1:1 orthologous genes using aTRAM across different species of lice**

**Suborder, species**	**Years**	**Contigs**	**Reciprocal**
	**Divergent**		**Best-BLAST**
*Anoplura, Pedicinus badii*	25 – 30^a^	1091 (98.6%)	1068 (96.5%)
*Anoplura, Haematopinus eurysternus*	65 - 70^a^	1089 (98.4%)	1048 (94.7%)
*Anoplura, Linognathus spicatus*	65 - 70^a^	1082 (97.7%)	1031 (93.1%)
*Anoplura, Proechinopthirus fluctus*	75 - 80^a^	1090 (98.5%)	1026 (92.7%)
*Ischnocera, Brueelia antiqua*	~110^b^	1102 (99.5%)	1060 (95.8%)
*Ischnocera, Columbicola liva*	~110^b^	1074 (97.0%)	1053 (95.1%)

Years divergent from the reference taxon were estimated in millions of years from a). Light et al. [23] and b) Smith et al. [24]. Contigs are the number of the 1,107 queries that assembled contigs in aTRAM. The final column has the number of contigs that passed a Reciprocal best-BLAST test against the entire *Pediculus humanus* protein coding genome.

## Conclusions

Overall these results suggest that aTRAM will likely prove useful for quickly assembling phylogenomic datasets across a wide taxonomic range. Furthermore aTRAM was designed to be agnostic to the type of input data and therefore future testing should include RNA-seq data as well as other types of markers such as UCEs.

## Availability and requirements

**Project name:** aTRAM

**Project Home Page:**http://www.github.com/juliema/aTRAM

**Operating system:** Unix, Linux, OSX

**Programming language:** Perl

**Other requirements:** Client needs free software including, muscle, mafft, blast, velvet or trinity

**License:** BSD 3-clause open source license

## Additional files

Additional file 1:
**DNA vs Protein query results.**
Additional file 2:
**Table of individual gene results summarized in Table** 2 **.**
Additional file 3:
**Fasta files of 3 genes (CO1, EF1a and one unknown nuclear locus), from lice in the genus** ***Degeeriella.*** Fasta files include aTRAM contigs as well as Sanger sequences.
