# Supplementary material for: aTRAM - automated target restricted assembly method: a fast method for assembling loci across divergent taxa from next-generation sequencing data
Source: BMC Bioinformatics. 2015 Mar 25;16(1):98. doi: 10.1186/s12859-015-0515-2 (PMC4380108; doi:10.1186/s12859-015-0515-2)
Supplement: Additional file 1: — DNA vs Protein query results. [file 12859_2015_515_MOESM1_ESM.docx]

**Additional File 1:**

**Allen et al., 2015 aTRAM: automated Target Restricted Assembly Method**

Aligned sequences of contigs assembled from the *Pediculus schaeffi* short-read dataset with aTRAM using either a DNA or amino acid target sequence from the reference sequence *Pediculus humanus*. Files indicate that the same or very similar contigs were assembled.

>EOG5B01CN_Protien_query

AGCAGTATATATATATATACTGCTTAAAAATTCTACAATTAATTGTAAAATAAAATTTTTTCTTTAGTTTAAAAATTTAAAAACATTGCTAAATTTTTCAAATTCTAATTTTATTATTAAAATGATCTGAAATCTGTTTATTTTAGTAACAATTAAATAAAATTGTTAACAGTCACAAATTTAATTTATTTTTCATTTATGCTAATATTTTAAAATAGTAATACTTAATAATTAGACTTTGGAAAAATATATAATTTAAATAATATCTTCAATTGTTAATATATAATGTTCAATATTGTTCAATATATAATTGTTCAATATATAATGGTAATAATGTCTTCAATTGTTGACATTTATCAAAAATTTAAATTTTAAATATTTAAAAAATTAGTGTATTAAAAAATAAAATTTTTATTAAATGAACAACTTTAATTTTTTTCACAGAGTAACATTACTTAATATGAGTTCTAATTTAAGTGCAAAAAAACAAACTACAACCTCAATTATGAAGAAAAAGGATTTAAAATCTGCTGCAAAAATCTCCAATAGTTTGCAAAATAATCAAAAAAAAGACATTTCAGTTTTTAAAACACCTACTTTAGGTACTAATAATGCTCATAGACATACTCCTGAAATATTTTTAACTCCAAAATTAAATACACCAAAAAGTTCTACTATGCTCAAAAGTAAATCAACTCCAGTTATACCTGAAAAATATTCAAACATAGTAAAAGGCAGAGACAGAGCTTTTGGTACACCTGTTACACATTTATGTTCTCCCAGTTTATCACCAAAAGGCAGAAGGAATTTTCTTCAGCAGGCAAACTGCTCTGTAAAAAGATTCCTTAGTGATTCTAGTTTAAATGATTCCCCTACTCCTAATAGTGGAGATTTAAATACTCCAACAAATAATGGTTATCAAAGAGATGAAGAGACCAGCAACTTTGTCGTAGGAGTCAGAGTAAGACCATTTAGTTTAAGGTATTGCATATTTTATAATTTCTTTCTTCTTTTATAAATAAAAATTTAATGATAATTTTTTAATTTATTTTTTTATAGGGAACTTAATGATTCTGCTGTAAGAAATGTTGTTCAAATGAAAGAGGAGGAAGTTCAAGTTACTTGTGATACAGGAGCTATCCACAGTTTTTCATATGACCATTGTTTTTGGTCTTTTGACAGTACTAATCTTCAGTATGCTTCACAAGAGGATGTTTTTAATGTTATGGTTTTGCCTTTAATGGATCAAGTTTTTTTAGGGTGCAATGCTTGCTTATTAGCTTATGGACAAACTGGATCTGGAAAAAGTTACAGGTTCTAAAAAATTACTTTTTTAAAAATGGAATTTATTCAAGTTTATTATCTAGTTTATTTAAAATTACTTATGACAAATTTCTCTTTTATTGAGCTTGCATAGAAAACTATACTTAAAATTTTATTTCAGCATGATGGGAATAGATTCAGGAGTCCAGGGAGAATTAGGCGAGGAATCTGGGATAATTCCCCGATTTTTTCAAGAAGTTTTCAGACGAATATCCGAAAGCGATAAAGAGAATATTGATTATCACGTTGAAATAAGTTATTTTGAAATATATAATGAAAAGATTCACGATTTATTAGGAACGCATTCAAATTTTTCATTAAGAAAAAGTCTTAAAGTGCGAGAACATCCTACTTTTGGGCCTTATGTAGTTGATTTATCTACTCATTGTGTGGAGACTTTTAGAGATGTTCAAGTATTATAAAAAAAAATTATTATTTATATCCGTAAAATATAATTATATAAAGTAAATTCTTTTTAAAACTATTGTTTAATGTAGGATTGGATAACTGTTGGCAATAGTCAAAGAATTACAGCTTCAACTGGTATGAATGAAAAAAGTTCGAGATCTCATTCAATATTTAGTATAACAATAACTCAAAAGCAAAGGGACGGACTTAATGCATGCAAAAGTAAAGTTAATTTAGTGGATTTAGCTGGGAGTGAAAGAGTTGCCCAAATGTGTGCGACTGGAAATAAACTTAAGGTGGGATTATTTTCTTTAAACTTTCAAAAAATAAAATTATTTAATATTAAAATTAAGTAACTTTACTAAGTTTTATTTTTATTCACCATTAATTTAGGAAGGTGTAAGCATTAATAAGTCTTTATTGAGCTTGGGAAAAGTGATAACGTTTTTGTCGGAAAAAGGTCGGAAGAAGGGTGTCACAAGTTTTGGGCCATACAGGGAATCGGTACTGACTTGGCTATTAAGAGTAAGTAATTTAAAAATTCTAATTTTTTTCCTGTAATTCAGTTAAGAATCAAAACACATTTCCCATAAGTGTTTTAAAAAAAAACACTGTTTTTATGCAAATTTATTATTTGTTAAAAATATTAAGGTTTAAAAGAATATGAAAAAATTTTTAAAGTTTTAATTCAAGTATTATATTCTAGATAATTTTTTTAAATGACAATAAAAAAAAATAAATTAATTATGAATTGTAAAAAGATACTGCATAAAAAGGTTTAATAATAGTTAAGTTTACAATTTTTAAAAAATTGTAAATTTCATATAATTTTTTTTGCATAAAAATTTTAAAATTTCAATAATTAAAGTTAAAAACAGTTTAGTGTGTATAAAAAACTATAATTATTGGTAATAATTTAATTTAAATTAAGGAATGTGTTTATATTAAATCGTATGTGTTTATTTCAAATGGGCAGTAATTGCGTATTCGATCTCAGTTAATTTTAATTGATTATTTATCTATCTAATTATAATATAGTAATAATTATAATTGATTAACTGTCTTATTATATCTATAAATCTTGTATTTAAAATATTAAAGAGACTCTTGGTATT-AAAAAAAAAAAAAAAATTAAAGCTAAAATGTACAAGCTGAAGAGTAAAAGAAGATTTTCAGGTTCGACTTGTAAAGTCAATAGAGCTGACTTTACAATTTCTTAAAATTAAAATAACTTATATATCTAAATTTGTTGTCAACAAATTTACATTCAGTGATTTTTTTTTTTTTTTTACTGTTTTACGTAAATTCTTGAGTTTCTTCTTTTTCTTCTCTTATGGTCGTAGATTTTTGACCTCCTTTTTTTTTATTTTTTATGTAATTGGATAAATTCTTTTTTTTGTTTTCTTGGCAAATGCTGCAAGTTTTCTCACTGACTTTTGCTTCTTGAGCAATTGCGTATTGCTTTTCTGTAAAATGTTTACATTAATTTATGGATTTTTTAAATTACGCGTTTTTTTTTTTTTTATTTACATTGAGTCACGTGTTCTTAAGAATATCAAATGAAAGAATTTACCTTGATGAATTTATTTTTATACGCAAATAAAAGTAAAGGGAAATTTCAATAAACTGGAAATATATTTTTCAATGTCAAGGTTGTTATGCACCATAAAATTTAATCTACAATTTTTTATAAATACATTACTGATTGAAGTGTAAAGAAATATTTAAAAATTAATAAATTTGTCTTCTGACGGCATTTTCATTAATTTTTTTATAATAATATTTTGTTTTTATTTTTGTTTCATTGTTTATTCGAAGGCTAACGTTATTACTTATGGCATTATAATAATTATATAATTCTTATTTTTTGAGATGGTCATTTCGCATCAGTGACTTTTTCGTTCTTTTATTTTTAAAACAGAAAAAAAAAATAAAAACAAAAATAGTTTT-----------------------------------------------------------------------------------------------

>EOG5B01CN_DNA_query

---agtatatatatatatactgcttaaaaattctacaattaattgtaaaataaaattttttctttagtttaaaaatttaaaaacattgctaaatttttcaaattctaattttattattaaaatgatctgaaatctgtttattttagtaacaattaaataaaattgttaacagtcacaaatttaatttatttttcatttatgctaatattttaaaatagtaatacttaataattagactttggaaaaatatataatttaaataatatcttcaattgttaatatataatgttcaatattgttcaatatataattgttcaatatataatggtaataatgtcttcaattgttgacatttatcaaaaatttaaattttaaatatttaaaaaattagtgtattaaaaaataaaatttttattaaatgaacaactttaatttttttcacagagtaacattacttaatatgagttctaatttaagtgcaaaaaaacaaactacaacctcaattatgaagaaaaaggatttaaaatctgctgcaaaaatctccaatagtttgcaaaataatcaaaaaaaagacatttcagtttttaaaacacctactttaggtactaataatgctcatagacatactcctgaaatatttttaactccaaaattaaatacaccaaaaagttctactatgctcaaaagtaaatcaactccagttatacctgaaaaatattcaaacatagtaaaaggcagagacagagcttttggtacacctgttacacatttatgttctcccagtttatcaccaaaaggcagaaggaattttcttcagcaggcaaactgctctgtaaaaagattccttagtgattctagtttaaatgattcccctactcctaatagtggagatttaaatactccaacaaataatggttatcaaagagatgaagagaccagcgactttgtcgtaggagtcagagtaagaccatttagtttaaggtattgcatattttataatttctttcttcttttataaataaaaatttaatgataattttttaatttatttttttatagggaacttaatgattctgctgtaagaaatgttgttcaaatgaaagaggaggaagttcaagttacttgtgatacaagagctatccacagtttttcatatgaccattgtttttggtcttttgacagtactaatcttcagtatgcttcacaagaggatgtttttaatgttatggttttgcctttaatggatcaagtttttttagggtgcaatgcttgcttattagcttatggacaaactggatctggaaaaagttacaggttctaaaaaattacttttttaaaaatggaatttattcaagtttattatctagtttatttaaaattacttatgacaaatttctcttttattgagcttgcatagaaaactatacttaaaattttatttcagcatgatgggaatagattcaggagtccagggagaattaggcgaggaatctgggataattccccgattttttcaagaagttttcagacgaatatccgaaagcgataaagagaatattgattatcacgttgaaataagttattttgaaatatataatgaaaagattcacgatttattaggaacgcattcaaatttttcattaagaaaaagtcttaaagtgcgagaacatcctacttttgggccttatgtagttgatttatctactcattgtgtggagacttttagagatgttcaagtattataaaaaaaaattattatttatatccgtaaaatataattatataaagtaaattctttttaaaactattgtttaatgtaggattggataactgttggcaatagtcaaagaattacagcttcaactggtatgaatgaaaaaagttcgagatctcattcaatatttagtataacaataactcaaaagcaaagggacggacttaatgcatgcaaaagtaaagttaatttagtggatttagctgggagtgaaagagttgcccaaatgtgtgcgactggaaataaacttaaggtgggattattttctttaaactttcaaaaaataaaattatttaatattaaaattaagtaactttactaagttttatttttattcaccattaatttaggaaggtgtaagcattaataagtctttattgagcttgggaaaagtgataacgtttttgtcggaaaaaggtcggaagaagggtgtcacaagttttgggccatacagggaatcggtactgacttggctattaagagtaagtaatttaaaaattctaatttttttcctgtaattcagttaagaatcaaaacacatttcccataagtgttttaaaaaaaaacactgtttttatgcaaatttattatttgttaaaaatattaaggtttaaaagaatatgaaaaaatttttaaagttttaattcaagtattatattctagataatttttttaaatgacaataaaaaaaaataaattaattatgaattgtaaaaagatactgcataaaaaggtttaataatagttaagtttacaatttttaaaaaattgtaaatttcatataattttttttgcataaaaattttaaaatttcaataattaaagttaaaaacagtttagtgtgtataaaaaactataattattggtaataatttaatttaaattaaggaatgtgtttatattaaatcgtatgtgtttatttcaaatgggcagtaattgcgtattcgatctcagttaattttaattgattatttatctatctaattataatatagtaataattataattgattaactgtcttattatatctataaatcttgtatttaaaatattaaagagactcttggtattaaaaaaaaaaaaaaaaattaaagctaaaatgtacaagctgaagagtaaaagaagattttcaggttcgacttgtaaagtcaatagagctgactttacaatttcttaaaattaaaataacttatatatctaaatttgttgtcaacaaatttacattcagtgatttttttttttttttnnn------nnnnnnnttcttgagtttcttctttttcttctcttatggtcgtagatttttgacctcctttttttttattttttatgtaattggataaattcttttttttgttttcttggcaaatgctgcaagttttctcactgacttttgcttcttgagcaattgcgtattgcttttctgtaaaatgtttacattaatttatggattttttaaattacgcg-tttttttttttttatttacattgagtcacgtgttcttaagaatatcaaatgaaagaatttaccttgatgaatttatttttatacgcaaataaaagtaaagggaaatttcaataaactggaaatatatttttcaatgtcaaggttgttatgcaccataaaatttaatctacaattttttataaatacattactgattgaagtgtaaagaaatatttaaaaattaataaatttgtcttctgacggcattttcattaatttttttataataatattttgtttttatttttgtttcattgtttattcgaaggctaacgttattacttatggcattataataattatataattcttattttttgagatggtcatttcgcatcagtgactttttcgttcttttatttttaaaacagaaaaaaaaaataaaaacaaaaatagttttaatttacttacgtaagaatttataattaggactgttttcttcccaaatcggataaacttttcgcattgaatacgagtgcattgccattgaatctg

>EOG5B01CW_Protein_query

TACCATATACTCAACAGTTGATACAACTTTAACACAAGTTATTTCACAATTAGAATTCTAAAAAAATTAAAATATAATATTGTACTGTAAACAGTCTGTTTTTTTAAAAATAAATTTTACTCGGTTAATAATGTACCTCTAGGCGTAGTCTTTGCAATTCACCACTTTCTCTGCAGTACCAATAAACTATACTTAAATTAGTACCCAGGACAATGTAATCGGAAATAACATCTAAACATGTTAAGTTTAAATTTTGCGAAAATAAACCCTTTTGAATTTTTAGGGGAATATGCTGCAGAAGTTGAGTCAGAGGAGACCACTCCTGCAGCAAGCCTTCATCTTCACAAATAGGTTCTGTCATTATTTAAAAACTTCAATTTTAGGAGTCATCGATAACGAATTTTTTAATGCTAGCTACCCCATAGCAAAATAAGTATTTTATATTACTGAAGAAAAGAGGCTCAACTTACTGTATTTGATTAGGCAGTTGATTAAATCATCGTGTAACCAGTGTGCACCCAATTTTAAATTTATTAGAGACACGTCATTAAATGCATTGTGTTAAATTTCTTATCGTAGGAGTTGTCAAGTTTTTCATTTTTGTAATTTTTTTATTTTATACTTATGGTTTATTTGACTATTAATTAATTTTTTTTTTACAATTTTTTAAAAGTGTATTTAATAATACAAACAATAGGTCAACATTTTTTATTAAATGAAAAATTTAACACAATAATAAAAATATTTTTTGTACTGAAACTTGTAATGTTAAATTCAACAAATTTTAAACGTTTTGTTTTTTAACTTTTTTCTTATACAACCCTTTGTAAATATTTAAGGTAAAATTATTCATGAAATTTACTTAATGATAAATAAAATACAAAATTTGTTTTTTTTTGATTCTTGTCTTAAAAATTATTTAAACTGTTATAATTAAACAGTGTTTTCGACGCTTTAGTATCCATATTTTTTTATAAAAAGAACCTTATTTAGTATTAAAATTTGTTAATTTGTAATTTTTAATATTAGAATGTTGAATTCGGATGAAACTGCTATTAATTCATTGTCAGCTGATAATGAAGTCACAACACGTCCTGTTAAAACTGGTGAAAGAATTCCTTTATTGTTAAGGAAAAGATTATTTAGGAATATTTTTAATTTTTCACATAGAAATGAAGAGAACAATGGGTAAGTTTTGAAATAAAAAAAATATTATTTTTACTAATTTAACTGTAAATACATCAAAAAATTTATTTTTTTTAGCTATGTTGAATTTGAAAACCATGCAGAAATTAATGGGAGGACATTAGGAAGTTTTGCAGGAGTATTTTGTCCAGTAGCTTTATCTATGTTCAGTGCTTTATTATTTTTAAGAGTTGGTATGATCAAGTTTTAATACTTCAAAATCTGATGTAATTTAATTATGTTAATATTATTTATCTAAGTATTTAAAACTTTTTGTTGCAGGATTTATTGTTGGCAATGCTGGTCTTTTATTAGCATTAACTCAATTTGTAATTGCATATTTAATATTAATATTCACTGTATCTAGTATTTGTGCAATATCAACAAATGGAGCAGTTGAAGGAGGCGGGGCCTACTGTATCCTTTTCATTTAAACTTTCTTTTTATTTTTTTTTCTCATGTTTTCTAAATTTCATATTTTGTTTCCTTAATTACCATTACAGTTATGATTTCAAGGACACTGGGACCAGAATTTGGAGGCTCTATAGGAACATTATTTTTTTTAGCCAATATTGTATCATCAGCACTTTATATTACCGGTTGTGTAGAAGGTTTTATTGAGAATTTTGGAACTGGGGGTAAAACAATTTTTTGTATAATAAAAAATAAATTCTATATCTTTTCCTTGCCAAAAGTAAATATAAATTATAATATAATTTATTATTTAACTTATTATTAATAATTTATTATTTAATTTTCAGGTTATCTTGTTGGTGGCAGTTCAAGTTTTGCTCTAAAAGATGGGGCTTGGTGGAGATTTTTATTTGGATCATGTTTAAACTTTTTAAATCTACTTATATGTTTGCTAGGGTCTTCAATATTTGCAAAAACCAGCGTATCTATTTTAGGAATAGTCTGCATATCTTTACTATCTTCCATTGTTAGTTTTCTTTGCACTGGTGCCTCTGAGGTAATCTCATAAAAAATATTTAAGTACATTTTGTGTAATAATAAAAAACTTTTTTTACAAAATAGACTTATTTTTAGATTCCAATTCCTATGAGAAATAATCTGGTGCAAAATGAAACCTTTCATGTAAATGGTACTTACACAGGGTTTAATTTAAATACATTAAATGAGAATTTATATGCTAATTATTCAAAAGACTACACATCTGGAAGTAATTCATCAGCAATAGATTTCGCTACGGTTTTCGGAGTTTTATTTTCAGGGGTGACTGGTATAATGGCTGGTGCAAATATGTCAGGTCAGCCTCTGCAATTATTAAAAACCACTATAATTTTATTAATATTTGTTTTTAACTCTAATTATTTTAAATTGAATATAATAATAAATACATTAGTCAATTTAAAAGATATCTTACAAAGTCTAAAGATTGGATTTTAATTTTAATTTACTTTTTATTGACTAAACAAAATAGCTATTTTCACATAATGACTTCATCTAAAATTTCTCTCTCAAATAACACAACGTAATAGTATTCTTAAAAAGTATTTATATTTCCTATAAAAAATAATATAAATAAAAAGTATTAGTTTTTAGCAGCCTCTTGCCATATTAAATTACAAAAAAAAAAAAAAAAATTATCATGTTATATTTTAAATTGGATCATCAGTATTTTTATTGATTTTTTTTTTTTATAGGAGAATTGAAAGAACCATCAAAAAGTATTCCCTTTGGAACTTTAAGTGCAGTTGCATTTACATTTTTTTGCTACATTATAATATCTCTGTTATCAGCCAGTACCACGAGTAGGTTTTTACTTCAAAATAATTACATTTTTTTAATGGGAATAAATTTTTGGCCTCCATTTATCACAATCGGGATTTTAACTGCAACGTTTTCTGCGAGCTTAAGTAATTTTATTGGATCTTCCAGAGTTTTGGAAGCTCTAGCGAAAGATAATGTTTACGGTAAAATTGTTGATTTGTACTGTAATTTTTTAAAAAATAAATTTAAGTTACCCCGCGAATGAATGATTTATAATAATTTTAATACTTTTAGGTTTCCTATTTAATTTCATATCAAAAGGAACGTATAAAGGAAATCCGATTGCTGCCGTTTTTGTGTCTTTTGCGTTGGTTGAAGTAATATTGCTCTTGGGAAGTTTAAATCTAATTGCACAAATTAACAGTATATTGTTTCTTTTGTCTTATTTAGCAACGAATTTAGCTTGTCTCGGCCTTGAAGTTGCAAGCGCGCCTAATTTCAGGTTCCTTAAAATTTTTATTGAAAAAAATTTTTTTTCATCTTAAAAAGAAGTTATTATATAATTAAAAAACTTTTTTTTTTTAGGCCGACATTTAAATACTTTTCGTGGCACACAGCATTTTTAGGATTGGCCTCGACAGTTATAATGATGTTTGTTATTAACTTAATTTATGCTCTGTTGAGTGTAATTTTATGCCTTGTTTTGATCATTTGCCTTCATTTATTTTCACCAAGTCGTAAATATCAGTGGGGTTCCATAAGTCAAGCTTTAATATTTCACCAGGTAATGAATTTTTCCCTAAATATGGGGGGTTTATTTTTAGCTCATGGCCATTTTTTTCAATAAATTTCAATTTGTTAAGGTGCGAAAATATTTGCTTTTATTGGATCCAAGAAAGGAACACGTTAAATTTTGGAGACCTCAAATACTACTTTTGGTCAACAACCCACGAACTTGCTGTCCTTTAATTAGCTTCGTAAACGACATAAAAAAAGGAGGATTGTACGTCATTGGGCACGTGCAATCGGGTTCTGATTTTTTGGATATCGCCACCGATACAACTATTGATGAATATCCGTATTGGTTGTCTTTAGTCGATAATTTAAAGGTACTTTCTAAAGTTGATTCACTTTTGTTTTAAACTTTTAAAATGATACACGCACTTACTGTGCCTACCATTGAAAATATTTCTATTTTTTTTTTTTTTTTTACCTAAAGGTGAAAGCCTTTGTGGAACTGACCGTGGCTAAAAATGTCAGAGATGGATTTCACCATTTGGTTAGAATATCTGGCATGGGTGCAATGAAACCTAACACTATTTTCTTAGGATTTTACGACGACGAAATGCCGATCGACTTCTTTGGAACGTGCGTAATCCCGTAAAATTTTTTATGAGCTTAATGAATATTTCGAATATCAGTGACCTTGTTTATCTCTAATCCGATTATTCAGTTTGTCGGTTTGAAAAATTTTTTTTTTTTTGCAGTGAAAACCCTTACAGTACAAAAGTTTTTCAAGAAATTGATATATTTCAATTAAGGAACATTAATAAGAAAAGTCTTACCATCGAAAATTATGCTGGAATAATATCTGACGTAATTAAAATGAAGAAAAACTTATGTATATGTCGCCGTTTTCAAATTTTGGAAAAAATAACTTCAAAGTGAGTAATGCCGGACTTTCGTAATTAATTCTTTTCTTAAATTTATCGGATTTCGTTTGCTTATATTTTGAAAAATAAAAATTTTCAGGTCTTCCAATACGTACATAGATGTATGGCCCATTAATTTTTTTTCGCCGAATGCTGATAATCCATTTGGAACGACATCTTTATTCATGTTTCAATTAGCTTGCATTTTGAATATGGTAAAAAAAAAAAAAAAATTTATTAAAATCAATTGTGAACGATTGTTTAAAAAACTTTAAAAAAAAAATAAAAAATAAAAATAAACATTAACATTTAAAGGTGTCAAGTTGGAAAAGATTAAAACTGAGAATATTTTTATGCGACAATAATTCGGAAAGTTTACCGGGAAGCGAAGCTCGTCTCAAGCAACTTCTTTTGTCTTTAAGAATTCAAGCAACAATTCACAGAGTAATAAAATTTAATTAATTCGAACTGTTTTTTTTTTTTTTTTTTTTTGTAATACATTAATTGATTGATTGATTTCATTAAATAAATTTTTTAGGTATTCGATTGGAGCAATTGTTATTCAATGTTACCCGGAGGATCAATTTATTCGGAAAACAATACCGAAAATAAGTGTAAACTTTATTTTAAAAAGTAAGGAGTTGGAAAATGTTAAAAACGTGTTATACTACAATCGGTTGCCCTTTAATTTTATAAATTTTCATTTTTCGCAGAATCAACGAGCTCATTCGAGAATTA-------------------------------------------------------------------------------------------------------------------------------------------------------------------------------------------------------------------------------------------------------------------------------------------------------------------------------------------------------------------------------------------------------------------------------------------------------------------------------------------------

>EOG5B01CW_DNA_query

----------------------------------------------------------------------------------------------------------------------------------------------------------------------------------------------------------------------------------------------------------------------------------------------------------------------------------------------------------------------------------------------------------------------------------------------------------------------------------------------------------------------------------------------------------------------------------------------------------------------------------------------------------------------------------------------------atttaataatacaaacaataggtcaacattttttattaaatgaaaaatttaacacaataataaaaatattttttgtactgaaacttgtaatgttaaattcaacaaattttaaacgttttgttttttaacttttttcttatacaaccctttgtaaatatttaaggtaaaattattcatgaaatttacttaatgataaataaannnn--------------------------------------nnnnnnnnttataattaaacagtgttttcgacgctttagtatccatatttttttataaaaagaaccttatttagtattaaaatttgttaatttgtaatttttaatattagaatgttgaattcggatgaaactgctattaattcattgtcagctgataatgaagtcacaacacgtcctgttaaaactggtgaaagaattcctttattgttaaggaaaagattatttaggaatatttttaatttttcacatagaaatgaagagaacaatgggtaagttttgaaataaaaaaaatattatttttactaatttaactgtaaatacatcaaaaaatttattttttttagctatgttgaatttgaaaaccatgcagaaattaatgggaggacattaggaagttttgcaggagtattttgtccagtagctttatctatgttcagtgctttattatttttaagagttggtatgatcaagttttaatacttcaaaatctgatgtaatttaattatgttaatattatttatctaagtatttaaaactttttgttgcaggatttattgttggcaatgctggtcttttattagcattaactcaatttgtaattgcatatttaatattaatattcactgtatctagtatttgtgcaatatcaacaaatggagcagttgaaggaggcggggcctactgtatccttttcatttaaactttctttttatttttttttctcatgttttctaaatttcatattttgtttccttaattaccattacagttatgatttcaaggacactgggaccagaatttggaggctctataggaacattattttttttagccaatattgtatcatcagcactttatattaccggttgtgtagaaggttttattgagaattttggaactgggggtaaaacaattttttgtataataaaaaataaattctatatcttttccttgccaaaagtaaatataaattataatataatttattatttaacttattattaataatttattatttaattttcaggttatcttgttggtggcagttcaagttttgctctaaaagatggggcttggtggagatttttatttggatcatgtttaaactttttaaatctacttatatgtttgctagggtcttcaatatttgcaaaaaccagcgtatctattttaggaatagtctgcatatctttactatcttccattgttagttttctttgcactggtgcctctgaggtaatctcataaaaaatatttaagtacattttgtgtaataataaaaaacttttgttacaaaatagacttatttttagattccaattcctatgagaaataatctggtgcaaaatgaaacctttcatgtaaatggtacttacacagggtttaatttaaatacattaaatgagaatttatatgctaattattcaaaagactacacatctggaagtaattcatcagcaatagatttcgctacggttttcggagttttattttcaggggtgactggtataatggctggtgcaaatatgtcaggtcagcctctgcaattattaaaaaccactataattttattaatatttgtttttaactctaattattttaaattgaatataataataaatacattagtcaatttaaaagatatcttacaaagtctaaagattggattttaattttaatttactttttattgactaaacaaaatagctattttcacataatgacttcatctaaaatttctctctcaaataacacaacgtaatagtattcttaaaaagtatttatatttcctataaaaaataatataaataaaaagtattagtttttagcagcctcttgccatattaaattacaaaaaaaaaaaaaaaaattatcatgttatattttaaattggatcatcagtatttttattgattttttttttttataggagaattgaaagaaccatcaaaaagtattccctttggaactttaagtgcagttgcatttacatttttttgctacattataatatctctgttatcagccagtaccacgagtaggtttttacttcaaaataattacatttttttaatgggaataaatttttggcctccatttatcacaatcgggattttaactgcaacgttttctgcgagcttaagtaattttattggatcttccagagttttggaagctctagcgaaagataatgtttacggtaaaattgttgatttgtactgtaattttttaaaaaataaatttaagttaccccgcgaatgaatgatttataataattttaatacttttaggtttcctatttaatttcatatcaaaaggaacgtataaaggaaatccgattgctgccgtttttgtgtcttttgcgttggttgaagtaatattgctcttgggaagtttaaatctaattgcacaaattaacagtatattgtttcttttgtcttatttagcaacgaatttagcttgtctcggccttgaagttgcaagcgcgcctaatttcaggttccttaaaatttttattgaaaaaaattttttttcatcttaaaaagaagttattatataattaaaaaactttttttttttaggccgacatttaaatacttttcgtggcacacagcatttttaggattggcctcgacagttataatgatgtttgttattaacttaatttatgctctgttgagtgtaattttatgccttgttttgatcatttgccttcatttattttcaccaagtcgtaaatatcagtggggttccataagtcaagctttaatatttcaccaggtaatgaatttttccctaaatatggggggtttatttttagctcatggccatttttttcaataaatttcaatttgttaaggtgcgaaaatatttgcttttattggatccaagaaaggaacacgttaaattttggagacctcaaatactacttttggtcaacaacccacgaacttgctgtcctttaattagcttcgtaaacgacataaaaaaaggaggattgtacgtcattgggcacgtgcaatcgggttctgattttttggatatcgccaccgatacaactattgatgaatatccgtattggttgtctttagtcgataatttaaaggtactttctaaagttgattcacttttgttttaaacttttaaaatgatacacgcacttactgtgcctaccattgaaaatatttctattt--ttttttttttttacctaaaggtgaaagcctttgtggaactgaccgtggctaaaaatgtcagagatggatttcaccatttggttagaatatctggcatgggtgcaatgaaacctaacactattttcttaggattttacgacgacgaaatgccgatcgacttctttggaacgtgcgtaatcccgtaaaa-tttttatgagcttaatgaatatttcgaatatcagtgaccttgtttatctctaatccgattattcagtttgtcggtttgaaaaattttttttttt-----------nnnnnnnnnntacaaaagtttttcaagaaattgatatatttcaattaaggaacattaataagaaaagtcttaccatcgaaaattatgctggaataatatctgacgtaattaaaatgaagaaaaacttatgtatatgtcgccgttttcaaattttggaaaaaataacttcaaagtgagtaatgccggactttcgtaattaattcttttcttaaatttatcggatttcgtttgcttatattttgaaaaataaaaattttcaggtcttccaatacgtacatagatgtatggcccattaattttttttcgccgaatgctgataatccatttggaacgacatctttattcatgtttcaattagcttgcattttgaatatggtaaaaaaaaaaaaaaaatttattaaaatcaattgtgaacgattgtttaaaaaactttaaaaaaaaaataaaaaataaaaataaacattaacatttaaaggtgtcaagttggaaaagattaaaactgagaatatttttatgcgacaataattcggaaagtttaccgggaagcgaagctcgtctcaagcaacttcttttgtctttaagaattcaagcaacaattcacagagtaataaaatttaattaattcgaactgtttttttttttttttttttttgtaatacattaattgattgattgatttcattaaataaattttttaggtattcgattggagcaattgttattcaatgttacccggaggatcaatttattcggaaaacaataccgaaaataagtgtaaactttattttaaaaagtaaggagttggaaaatgttaaaaacgtgttatactacaatcggttgccctttaattttataaattttcatttttcgcagaatcaacgagctcattcgagaattatcagatagaacttcttgtttgttcatgtatctaccaactgaattttacgaaagtcaggacgcttccaatcacaaaacttatctacaatatttgacggatttaaccgaagaattaccgcccacggttttcgtacacggcgttcacggcgtcactagcacgacgctctaaaagaaaaaaaaatacataaataataataataatatttttaaaatgattttaagattcgaaacgaacagtattttatcgaaaatttcgattcacaaaaacaaacgaacgtaaaaaaataaaaaacatatcctccgaacgtacgaatcgtctacgcctacgccgaaatcgaaaacaacaaacaaaacaaacaacggagagatagaaaaatttttagagaaatcaaaacgatccgctcgccttaaaatattttctactagtatgaattacgcgtttaacgtttaattaacatatcgaaaattcaaatcgacagaaa

>EOG5B01DZ_Protein_query

GATAAAAAAAATATTTTTTTTTTTTTCGAAATTCAAAGTTAAAAATAATAAAAATTAATCCTTACCTGATTTGAACGTAAGAAAAAATTAATAATAAAAAAAAGAAAATATTTATGATTCGAAGATTTTTTTTTTTCTTAACGTTGTCAGTATTTATCACATTTAACAATAGTAATGCACTTATATCATTGATAACCTTTTAAGCGGCCGAATCGATTTTTTTTTTTTTTTAAAGTCTTCAACATTCGTCCGGTTTTGACGTCATCTAAAACCGTTGAAAAATGATACCGTAATATATTTTAAATTAAAAAATTTCACTACTGTAAATAAATAAAACGTGAAAAATATAATTGAAATATTTTAAAATTTAGCCTGTTGTTCAGACTGTATCACCTCGTACAAATGCTCGCGAAATTAAATCGTTTATTCACTTCGAGTTACGAATGACGTGCGAATCGTTGTGTCAGTGAAAGTTGCACTATTATATACACTATGATAACTAAAAATTTTATTTGAAAATAATTCTTGTGACACGATGAAGAAAATTATAAAAAAAAAGTATTGCATTTTTATTAAAAAAATAAATTATATTTTAATTGATTGAAAGAAGCCAACTAACCATAATTAAATAATTTTTTAATTAAAAATTAACGGACAAGTAGTAATAATGATGTGAATATAGAAATTATAAAATTAAATACCGTCTGCTCAAAATAAAAACAGGGCTGACAACAATTTTTTTTTTTAAAATAAAAAATGAGTAACAATTAATTTTTATGAATCACATTAAATGATAATAAAAAGTTAATTATACTTTACTAAAATTCATTTCACTATTTTAAAAAATTTAATAAAGAAATAATGAAAATAATTTATCTATCTATTCTTATGTTTGGAATTTATAAGTTTTTAAATTATAAAATGTCAAAATAATATTTTAATTTATAAGAAATAAAAAAAAATTCGAATTGTCACGATTGACGCCAAAATTATTTTAAAAATGTTTAGAGTTACGTTGGCTTATGTTGGCTGTACTGCAATAAATTTTAGAAAATGGAGGAAAAAGTAAAAGTTGTTGTCAAATGGAGTGGTAGAGAGTTTGAAATATCTAATTTATCACCGAGTGATACCGTTGAAAAATTAAAAGATGCTATTTTCAGAGAGACTGGTGTACGCCCTCAAAGGCAAAAACTTCTTAATCTTAAATATCGAGGTAAATTTATAATAATTAGAAAAATATCTCAAACTTACTTTATCAAAATGTTATCTATGTCTAACTTTAAAAAACTTCTATTTAATTTATCCGTTTATTCACTTAAAAAAAAAAAACCCACCTATTAGTAGTTTTAACTTCCAATACTTACATTAATAAGATATTGTGAAAATGAAACCTTATGGTTAACTTATTGTTAAAATATTTTTTTCGCAAATTTGTTGAAATATTATTTGTATAATTTATAATATACTATAATTAAGTTTATTATAATAAAGTAAATAACTTACTGCCGTGATTGAGTAACTATGAATTCATTTGCGTATGTATAAAATTCTATTTAAAAAAAAAATTACTATTTTCCATATTGTAGCTAAGCCTGCTGAGGACTCATGCAAAATGTCAGCATTAAATTTGAAACCTGGTTTTAAAATAATGATGATGGGTTCTCTCGAAGATGATATAACTCAAGTCAACACAATTCCACAAGGAATACCTGATGTGGTCAATGATCTAGATATTGAAGATGCAGAAGTTGCTATAGAAAATATGGAGGTAAGAAAACCATTCAAACTATTTAATTGTTGATTAGTAATCCAAATTGAATTTTCAGAAAATTCCAAATTTTGTTAGAATAACTTCATAACTTTTGTTAAAGTAAAAACAATTTTTTCGCTAATATTTTTTCTTATTCATTATTAATTTTAAATTCATATCATCCAATTTTATAATTAAGTTTGTTTATTTCTTTTCTTTTGCATTTAGCAAAAATTAATAAATTATCCATCATAAAACAAAATGCACTAATGTAATATATGCTTTCAGTTTTATTTTTTTAGTGTAGTATAATTTTCTGCAAACTTAGAAAAGTAAGTAATTTACAATGTTAAGTGAAATATTAAATACATCATTTAAAAAAAAAAATTGTGATAGCATTTGTTTTTTTATTTAAAATCATTCTCATTGAAAACTTCATGCAGAACTTTTTATAACTACAAAAATTATATGTGTTTAATGCATAATGAATTTTTAAAATAATTATTTTATTTGGAACTCTAACTTTCCTTTTGATTTAGGTACATCTTTCTAAAATAGAAAAAAGAATAAAAGAATATGAAATTACAGAAAGGAGTGAACCAAGAGCAGGGAAAAACTTATTGGTTCTAGATATTGATTACACTTTATTTGATCATAGATCTGTGGCTGAAAGTGGAGCAGAGCTAATGAGACCTTATTTGCATGAATTTTTAACTTCGGCGTATGAAGTAAGATTTTTTTTTTCTGAAAGCGTTGATAATTTAAAAGATTTTTAAACAGTTTTATAAATTTTTTTTACTTTTAGGACTACGATATAGTTATTTGGTCGGCAACAAGTATGAAGTGGATTGAAGAAAAAATGAAATTGTTAGGAGTTTCAACTCATACAGATTATAAAATTCTATTTTATCTCGATAATCTGGCAATGATATCCGTTCATACTCCCAAATATGGCGTAATTAACGTTAGTCTTGC-TTTTTTAAAAATTATTTAACAAAAAAAAAAAAAAAATTCATACAATGTTACAAACTTATTTTAAAAAATTACAGGTGAAACCATTAGGATTGATTTGGGGAAAATATCCACAATATTCAACGAAAAATACAATTATGTTTGACGACATAAGAAGGAATTTTATAATGAATCCTAACAACGGTTTAAGAATCAAAGCATTTAGGCAAGCGCATTTAAACAGGCAAACCGACACG------------------------------------------------------------------------------------------------------------------------------------------------------------------------------------------------------------------------------------------------------------------------------------------------------------------------------------------------------------------------------------------------------------------------------------------------------------------------------------------------------

>EOG5B01DZ_DNA_query

-----------------------------------------------------------------------------------------------------------------------------------------------------------------------------------------------------------------------------------------------------------------------------------------------------------------------------------------------------------------------------------------------------------------------------------------------------------------------------------------------------------------------------------------------------------------------------------------------------------------------------------------------------------------------------------------------------------------------aaataccgtctgctcaaaataaaaacagggctgacaacaatttttttttttaaaataaaaaatgagtaacaattaatttttatgaatcacattaaatgataataaaaagttaattatactttactaaaattcatttcactattttaaaaaatttaataaagaaataatgaaaataatttatctatctattcttatgtttggaatttataagtttttaaattataaaatgtcaaaataatattttaatttataagaaataaaaaaaaattcgaattgtcacgattgacgccaaaattattttaaaaatgtttagagttacgttggcttatgttggctgtactgcaataaattttagaaaatggaggaaaaagtaaaagttgttgtcaaatggagtggtagagagtttgaaatatctaatttatcaccgagtgataccgttgaaaaattaaaagatgctattttcagagagactggtgtacgccctcaaaggcaaaaacttcttaatcttaaatatcgaggtaaatttataataattagaaaaatatctcaaacttactttatcaaaatgttatctatgtctaactttaaaaaacttctatttaatttatccgtttattcacttaaaaaaaaaaaacccacctattagtagttttaacttccaatacttacattaataagatattgtgaaaatgaaaccttatggttaacttattgttaaaatatttttttcgcaaatttgttgaaatattatttgtataatttataatatactataattaagtttattataataaagtaaataacttactgccgtgattgagtaactatgaattcatttgcgtatgtataaaattctatttaaaaaaaaaattactattttccatattgtagctaagcctgctgaggactcatgcaaaatgtcagcattaaatttgaaacctggttttaaaataatgatgatgggttctctcgaagatgatataactcaagtcaacacaattccacaaggaatacctgatgtggtcaatgatctagatattgaagatgcagaagttgctatagaaaatatggaggtaagaaaaccattcaaactatttaattgttgattagtaatccaaattgaattttcagaaaattccaaattttgttagaataacttcataacttttgttaaagtaaaaacaattttttcgctaatattttttcttattcattattaattttaaattcatatcatccaattttataattaagtttgtttatttcttttcttttgcatttagcaaaaattaataaattatccatcataaaacaaaatgcactaatgtaatatatgctttcagttttatttttttagtgtagtataattttctgcaaacttagaaaagtaagtaatttacaatgttaagtgaaatattaaatacatcatttaaaaaaaaaaattgtgatagcatttgtttttttatttaaaatcattctcattgaaaacttcatgcagaactttttataactacaaaaattatatgtgtttaatgcataatgaatttttaaaataattattttatttggaactctaactttccttttgatttaggtacatctttctaaaatagaaaaaagaataaaagaatatgaaattacagaaaggagtgaaccaagagcagggaaaaacttattggttctagatattgattacactttatttgatcatagatctgtggctgaaagtggagcagagctaatgagaccttatttgcatgaatttttaacttcggcgtatgaagtaagattttttttttctgaaagcgttgataatttaaaagatttttaaacagttttataaattttttttacttttaggactacgatatagttatttggtcggcaacaagtatgaagtggattgaagaaaaaatgaaattgttaggagtttcaactcatacagattataaaattctattttatctcgataatctggcaatgatatccgttcatactcccaaatatggcgtaattaacgttagtcttgcttttttttagaattatttaac-------aaaaacaagttc-----atgttacaaacttattttaaaaaattacaggtgaaaccattaggattgatttggggaaaatatccacaatattcaacgaaaaatacaattatgtttgacgacataagaaggaattttataatgaatcctaacaacggtttaagaatcaaagcatttaggcaagcgcatttaaacaggcaaaccgacacggaacttctcaagttgtcgcaatatttaaaagatattagcatgtgtgaagatttttctgaactcgatcacagacattgggaaaaatattcaaaaaagaaaagggaaggcaaaatatgaaaatgggtgtgttattattnnnnnnnnnnnnnnnnnnnnnnnnnnnnnnnnnnnnnnnnnnnnnnnnnnnnnnnnnnnnttttttttttttttttccctcgcgtcgatcggaaatttttttttgcaaaaacatcaatcaaatcgttcgacgatgaatgaataaaactcaaaaaatttgttcgcttgttaatgtagcaaaaataaatgaattgaaattttacgttgcaaatatattatttcattttcaaatattgtttatagagtaatactacttctcaccaagtaacgtatagaaattgttttgctgcattatcaataacggaaataaatacatatgaaatttttacatttagttaaatgtaaaatttt

>EOG5B01F8_Protien_query

AAAATATGAAGAGGCCGGCACTAGAGTGAACAACGTAATGTATAAGAAAATTTACTCTATAGAATTATTGTTTTTGACGTATTACAAAAAAAATTTTTTTTTATTCAGATTTCAAAGATTTTTGAAAAAATTGATTTGATAGTCAAAATTAGTCAGTTTTTAAACCTTTTAGTGTTTTTGGTGCAAGGAAAATATCCTACAATAAGTGATCGATTGCTAAAATTAAAATATATTCCAACTAGAAAGTATAATAGAAGTGTTGGGTATCATTTTATGACTAGGCAACTTGTATGGTTTAGTATTTTGGTTTGTTTCTTTTATTTTTATTGTTCATTTCATTTAAACAAAATGTGTAATTTAAATGAATTTTTTTTATTTTTTATAGGAGTTATTGAAATTAATTTTACCACTAATTAATTATTCGAAAATTAAAAGCAAAATTAGTACCTTAGTTTTTTCTAAATTTTATACAAAAAGCATTAAAATTCATACTAAGTATAAAAATAATTCCAAATGTGTTATTTGTAATGATTATTTAATTTTACCATGTACAGTATCCAAAAACTGTTCTCATTTAGCTTGCTATTATTGCTTAAAGGTAAAATAATTTTTAATTTTAAAAAATATGATAAGAAAAATAATTTAAAAAAATTTTTTTTTACAGAGTATTATTTTATCAGATAATAAATATACATGTTCTTATTGTCAAGAAACCAATACTGATTTAGACACTTTTCATTTTTATTCCATATCTGAAGAGAAATAATTGGATTCTAATACAAAAAATTAAGAGGTATAATTGATGCTTTAAATTTTATAAAAAATTATTGTGTTATTAATTTTTTAAAATCAGACTTATAAGAAACTAAAATAAGGTTTATTTGTTTTATTATTAAAAGTTTATTTTTTAATTATGTAATTTTTTATTTTTAAAACCCAATTGACAATTGCCTTTATCTTTTATTTTAAAAACACTTTCTTTTTCTGAAATTCAAGGGTGTCAATTTTTTATTTTTATAATGAATTTAATAATTTTTTTTATAAAATTAATAATAATCTATATAATATAAATTAATAATCCGTAAATTTTTTCCTTTTTGAAAATTATTAATTTATTTTGCTTATGTATACCTTTTATACATATTTTATAAATTTTAAAATATAATGTTATTTGATAATTAGAAAAAAAATTAATTTTAGTTCTGTAAAAAAAAAAAAACAAAGAGTACGTATTAATATACACGAATTAAAACCGTCAAAAGGCTTTGAATGAAATCGTAGTGATTAATCTCTTTTCAAAACCCACTGATAAGAGAATTAAGATAAATATTTTTAATTTTTTTTTACGATGGTAAACAATATAGGTTGCGATTTAGGTAATTATTCAAGTTTTGGAAATATGTAAATTTTAAATGATCTAATATATTTTTCTGTTTTAGATTTAGGTTGGATTAATGGCGTATCTATTAATGCTTGGTGTTTATATGACAATGAAAAGGATGTAAGCCAAGAATGTTATAAACTAGACGGTATATATAAAGCAGCATGGCTGTTAAAAGCAGTTTCTTGTTTAGATCTCACAACCTTATCTGGAGATGATACTTGTAAATATTTATTAAGTACATTTATTTGTTACTTATACTTTTTTTTTTTTTTAATTATCATTTTCTTTTATTTAGGGAGCAATGTAAATAGATTAACAATAAAAGCTTTAAATCCCATATCATCGAAAATATTAGATGCACTTGAATTTCCTAACAAATTATTTACTACAGCTGCTGTTTGTGTGTATCCCTCTAGAGTTATTGATGTAGTATCTACCCTAAAAAAAGTATCACTAGTTGAAAAATTACCTGTTGCTGCTGGTAAATTGTTAATAGTACTTTTTTTAATTGTAGTTTAAGAATTTTTGTTGTATTCTTTAATTGTTGTAAATTGTAGTTAGCACTGGTTTTCCCACTGGACAGTATTGTTTAGCATCCAGACTTCATCAAATTCAAGATTGCATAAATAAGGGTGCCAGTGAAATAGATGTAGTTATTGATCGTTCATTAGTTTTAACTAACCAATGGGAATTATTGTACAAGGAATTAAAGCTTATGAAGGTTTTTACAAGTATTATAATATTTAATATTAATAATTAAATTTCATGATACAATTTTTAAAATATATCTTTATTAAACTTAAAATATTTTAGGAAAAGTGTAGGTCTGCAAAAATGAAAACAATATTAGCTGCTGGAGAATTAGGCAATTTTAGAAATGTATACAAAGCTAGTTTAGTGGCTATGATGGCTGGTTCAGATTTTATAAAAACTTCAACTGGAAAAGAACCTATTAATGCTACCTTTCCATTAGGCACTGTCATGATGAGAGCTATTCAAGAATATTATAAGAAAACTAACTATAAAGTAAAATTTTAAAAAAAATTTAATTATTTTTTAAATAAAACATTCTATTTTTGAAATTTAAATTAATTTTAGGTTGGTTTTAAAGCTGCTGGAGGAATAAAAACTTCAAATGATGCATTAAATTGGTTGGCATTAGTGAAAAAAGAGTTAGGTATAGAATGGCTTAATAAATCATTGTTTCGAATTGGTGCTTCTGGACTCTTACAAGATATAGAAAAGCAGTTATATCTTTTAACTTTTAATACAGTTCCAGCAATAAATGAATTTACCTTGGCTTAAATATTAAAATAAAAAATGAATAAAAATATGTACAAAGGCATGTGTAAAAAAAAAAATTACAACTGGTATATAAATTTTTTCAGTTGCACATAGTTAAGTTACTAAAAAACTTATTCAATAATTTTATTGCCTTTTTTTTTGTAAATAAAAGTTTTTCTTTTTAATATCTGAAATTAAATTAGTTTTTCACTGTCACCCTTGATCCATTAAAAACCATTTTTAAATTTAAAATATTCAAAGCCTTTTTATTATTTGATTCTTGATCAAAAATTTTTTCAATAATAATTTCAATGTCTACTGAGGCTAATTGCTTTTCAAAGAAATTTTAATTTATCATCTTTTAGGTCAATAGGTTGCTAAAAATTACATCAACATTTTTTTAACAATGACTCATTATCAACTATTAAGAATCTAATTACAAGTAGTTAAACAATAAAAAAAAAAACAATTAAGTTTTCTGCTAAATTCAGTAATTTAATATTAGTTCAATTATCTACTTGCAACGATTAAATTAAATAATGTTAATTGAGAACTTTTTAAAACGAATTTATATGCACCAGAGGCTTTTAGTTATTCTTTTTTCACATAAGAAAAGATTAATATAAAATGAAAGAGATCAAAATAATGATTTATTTATAAAGTCCAACATATTAATTTGATTTAAATTTACATAACAACATTAATTAATATTTTAAATTTCCAATGTATACAAACTAAGATTATTACTATCTTCGTAATTAAATTAAAAAATGACTAAATCTTAATATGCCGGCTTTTGTTTGGATAAAAGTTCAATTCCAGCACATAAAATAAATTTTCTCCAATAGAGAAATCGTAAAAAAAAAAAAAAAACGTTGATTCAACTATAAAATTATTCACTTACTTTCATAACATTAATTTTAAAAAAATAATAATAATAAAATAATTGGCTTATCTAAATGAGAATAAAATAAATTTGAAATTAAAATTATTATAAATGTTTTTACATATGTCTATTATTTAATTCTTAGAAATATCACCAGAATTAAATTATATATAAAAATTTCATAGGCTTCTAAATACATAACTAATACATTCTATATATTTAAAAGTCCATATGTTTCTCCTTCATCTGATGATACATCAATTTCTTTTTTACAAATAACCTTCTCTTTCTCACCAGTCTTTAAAACATCATTTTTATCACAGCCTACATTTAAACTACAATGTGTGCTAAGAACTTGTAAAGTCCTAAATTTTATGTCATTATTTTTTAATCCATCATCTTTACAATTTATATCAGATGTAGATAGTAGTGAATGTGATGGAACATTAAGAATTGCTGAATTAGTTTTCGGGCAAATGTATACTTGAGGTGGAGCTTCAATTTTTGATGACTGCAATTTTGAGAGGTCTGAAGGCAAAGCTGGTTTTTCAGG

>EOG5B01F8_DNA_query

-------------------------------------------------------------------------------------------------------------------------------------------------------------------------------------------------------------------------------------------------------------------------------------------------------------------------------------------------------------------------------------------------------------------------------------------------------------------------------------------------------------------------------------------------------------------------------------------------------------------------------------------------------------------------------------------------------------------------------------------------------------------------------------------------------------------------------------------------------------------------------------------------------------------------------------------------------------------------------------------------------------------------------------------------------------------------------------------------------------------------------------------------------------------------------------------------------------------------------------------------------aaaaaaaaaaaacaaagagtacgtattaatatacacgaattaaaaccgtcaaaaggctttgaatgaaatcgtagtgattaatctcttttcaaaacccactgataagagaattaagataaatatttttaatttttttttacgatggtaaacaatataggttgcgatttaggtaattattcaagttttggaaatatgtaaattttaaatgatctaatatatttttctgttttagatttaggttggattaatggcgtatctattaatgcttggtgtttatatgacaatgaaaaggatgtaagccaagaatgttataaactagacggtatatataaagcagcatggctgttaaaagcagtttcttgtttagatctcacaaccttatctggagatgatacttgtaaatatttattaagtacatttatttgttacttatacttttttttttttttaattatcattttcttttatttagggagcaatgtaaatagattaacaataaaagctttaaatcccatatcatcgaaaatattagatgcacttgaatttcctaacaaattatttactacagctgctgtttgtgtgtatccctctagagttattgatgtagtatctaccctaaaaaaagtatcactagttgaaaaattacctgttgctgctggtaaattgttaatagtactttttttaattgtagtttaagaatttttgttgtattctttaattgttgtaaattgtagttagcactggttttcccactggacagtattgtttagcatccagacttcatcaaattcaagattgcataaataagggtgccagtgaaatagatgtagttattgatcgttcattagttttaactaaccaatgggaattattgtacaaggaattaaagcttatgaaggtttttacaagtattataatatttaatattaataattaaatttcatgatacaatttttaaaatatatctttattaaacttaaaatattttaggaaaagtgtaggtctgcaaaaatgaaaacaatattagctgctggagaattaggcaattttagaaatgtatacaaagctagtttagtggctatgatggctggttcagattttataaaaacttcaactggaaaagaacctattaatgctacctttccattaggcactgtcatgatgagagctattcaagaatattataagaaaactaactataaagtaaaattttaaaaaaaatttaattattttttaaataaaacattctatttttgaaatttaaattaattttaggttggttttaaagctgctggaggaataaaaacttcaaatgatgcattaaattggttggcattagtgaaaaaagagttaggtatagaatggcttaataaatcattgtttcgaattggtgcttctggactcttacaagatatagaaaagcagttatatcttttaacttttaatacagttccagcaataaatgaatttaccttggcttaaatattaaaataaaaaatgaataaaaatatgtacaaaggcatgtgtaaaaaaaaaaattacaactggtatataaattttttcagttgcacatagttaagttactaaaaaacttattcaataattttattgcctttttttttgtaaataaaagtttttctttttaatatctgaaattaaattagtttttcactgtcacccttgatccattaaaaaccatttttaaatttaaaatattcaaagcctttttattatttgattcttgatcaaaaattttttcaataataatttcaatgtctactgaggctaattgcttttcaaagaaattttaatt-------------------------------------------------------------------------------------------------------------------------------------------------------------------------------------------------------------------------------------------------------------------------------------------------------------------------------------------------------------------------------------------------------------------------------------------------------------------------------------------------------------------------------------------------------------------------------------------------------------------------------------------------------------------------------------------------------------------------------------------------------------------------------------------------------------------------------------------------------------------------------------------------------------------------------------------------------------------------------------------------------------------------------------------------------------------------------------------------------------------------------

>EOG5B01FP_Protein_query

TCTTCTTATGTTTTATTATATATTTAAATATAAAAACTATACTTTTATAAAAATAGGTTTAAAATAACCTTGGTTATTATGTCTTTTTTGTTTTGTTTTGTTTTTTTTAAGCCTTAAGTTTAGTGTATATTTTACATGAGTGAAATGTTCAGTTGACATAAGCATTTTATTATCATGAATTTAAAACTGCATATATACAAGTTTTCGAATAACGGTATAATTCTTTAAAAAAAAAAAAAAAGTCAGCTTACTCTGACATTTTCTTTTATTAATTTTTGCCTGACATTTTTGCATTAATGCAAATCAATAGTTGTGTTATAAAAACAGAAAAAACACTGTTTTAATAAAATTATTTTATACGCGAACTTAAAAAAAATAAAACAAAACTAAAAAGGAAAAAAGATAAAGAATGAAAAGTTTCAATAAAGTTAAAAACGTGTGAAAAAAAAATTTTTTTCACAATCAAAATTAAAAGTGACCCTATTTAAAATGGCGGAAGATGAAAATTTATACACCGAAAAACCGTTAACAATTTTTAAGTAGATTTCAATTGTTCAAATGTGTTTAAAAAAAAAATATTTTGAACTTATGTATAAATGAATAATAAACGCATTTTTTATTTAAATATTTATAAGTTAAAATTATTTTCTATTCGGTAAAATTAGAAACAATTTAAAATTAGAATTTTTTTTTTTTTGAATTTTTCAATTCATCAATATGTAAATTATTAACAATTTACTAGAAATTTTATTTGCATAAAAGTATATCTCATGCAATTAGCAAAAAATAAAGACTTATTTTAAGAAAATTAGTCAAAAATATAACTAAATATAAAATTTTTTTTAAAATTCAAAATAAAATTGCTCAATTTTTACAGTTAAAAGAAAGATCATGTTTGCCGAAAAAAACTGACAAAAATGTGCTTGAAAATTTAATTTAACTTTTAAATATTTAAATGCTACTTATAGTTAAAATAAGTTTATTGCTTTTTTTTCTTACTGTTCCGTGTTTCTTTCAATTACCTCGTAATCTTTATCTGGCACAATTTTTTTTTTTTTTTAAATTGACGAAATAAAAATACAAGTTTCTAAACTTTTTGCTGTTATTCAATAATTTTGCTGTAATTTATAAAAACAAAATGTCGCTTTGGAAGTCTTTAAACCGTTTATGTTTACATTACAATAATTTTAGTAAGAGAATTGTCACAAAAAATATTTTCAATAATGGGTAATTTAAATACTATGAATTTTATTTAGTAAATAATTCATCATACTTCACTTACTTTTCGAGATTTATTAGTTTGCGCATTAGATAAAAGTAAATTAAACTAAAAAAAATTTTACATTTTAGATGTCGTACTCCTATCATTCATCCGACGATTTCAAACCGTCCTGTTCATATTTCTAGTATAAATAATGATATAATGGAATTTTTTGATGATAAAGAAAATTGGGGAGCTAGAGAAGTTTCAGTTGGTAGATCATGGAAAGTAGAGGAACTTAGAATTAAATCAAATTCTGATTTACATAAATTATGGTACATACTATTAAAAGAAAAAAATATGTTACTTACCATGGAACATGAATATAATGTAAATACTGAATTGTAATTTATATGTTTAGTATTATTAAATTAAATGTTGGTACAAGTTCTTTTTTCTTGTATAGAGATTGTATAAAGCAATGCCAAATCCTGAAAGAATTGATAAAGTTGAAGAAAGTATGGAAAATTTAGAAAAAGTTGTCAGAGAAAGAAATCGAGCATATTATGAACTTGAAACTGGAAAAACAGGAGAACGCCCTTTCTGTGATAAAACAAATTGTTTTGGTATAATTTAAAAAAAATTTATTTAATAAAAATATTAATGATAATTATATTTTACTATTGTAATACAAATTTTTAAATAATGTTGTAGGATTAACTTACAAGTACAAAATGAACCAGTATAAAGTACCACCTTATTTAAATAAAAAGTATTTGGAAAAAGATAAGTTAGAAAATCAAACAGATGATCATGTGTATATTCAGGACTTTTTAACAAGACTCAAGGAAAAAAAAGCTAAAAGAGATCATTATCACAGAAGGTATGATAATTATAACTAATTTTTTCCATAAACCATTAGTTTCTATATTTTATAAACTGAAATTACTGTATATATTTTAAAAGTGTCTTGGGTATAAATTTGTTCAATGCTAAAATGAAATTTTTTAAAAAAAGTATTACTGTGCTCTTCTTTATACTTAAGTAATTTTAACTTAAATATAATAATGAAAATGATTATTGCTGTCAGCAAGCATATTTTTTCTGTTTTTATATCAATATAATAATAATTACATTAATTGATTTTGTGGTACATAAAGATATATGTACATATGTATTTAAAATTTAATATTAATTTCAGAGGAAGGTATATGGTTGTTTGTAACTTGCTTAAACGGTTTCCTGATTTGGATGAAGAAGCATTAAAAGAGCGATTTCCAGATGTGAATATTGAAGCAGCTAAAAAAGATAAAAATTCAAGAGGCAATCGCGAAAATCCTTATTAAAGATATTTAATTTTAGGAAATTAATAAAATTTTTTAAAAAGCACATTAGTTTTTTTAAATAATTATTAACATGATCCATTACGTACGATTTACCAAAATAAATAAGTTTATAATAGAAAATCTTAAATTCAGGAATAATTCATTACATTTTTGGTAATAGCAAAATAGTCGTAAAAAGCTTATTTTGCTACCAATCAAAAACCAAATGGAAGATAATTTTTGCTTAAATTAACAGTAGTATTTGTACCATGAAAAAATGGCGGATGAAAGTGAGAGTTATCCATTGCACGAATGCGTTTTTAAAGGTGACGTGAGAAAACTATCTTTATTAATTAGATTAAATGACATTAGCAAGAAAGACAAGCATGGTAAGTAAATCTTAGTACAATTTTTAAAATATGTCGCTTTGCTTAACACTACAGTCACATCGTAAGATATTTCTATTTTTGGACGTAATTTGACATTTCTAAAATTCACGGGAATATTTTTCTATTTATTTCATTTTATGACTAGTGTGCTTTAATAATTTTCTGTACTCAGTTTTAAAAATAAAGCTCCTAAATTAAAAGTTTTAAAAAAATTTTTTTTTAAATAACAATTTTTTTTATCAACAGAAAAGTTATCTTTTATAACTATTTTCCATTACCTGTAAATATAAAAAAATAAATTTTTTTTTTTTTTAAATTTTCTAGAAGTTGTCTTAATTTACATTTTCATTTTTTTTTTTTTTTTTAGGAAATACAGCATTACATTTGGCAGTTATGCTTGGAAGAAAAGGTTATTTTCTATTTTCTTTTCTAATTTATTCATAATGATGGTATGTTTTTTAGGTTATAATTTAATAATTTACTTAATTTTTAGAATGTGTCCAATTGTTGCTGGCTCATGGAGCTCCAGTTAAAGTAAAAAATTTGGCTGGATGGAATCCCCTAGCTGAAGCTATTAGTTATGGGGACAGGCAAACCAGTAAGATCTTTTTGGATAAATATTACATACATATATATGTTTAAATAATTATAATTATCATTTATAAAACATT

>EOG5B01FP_DNA_query

-----------------------------------------------------------------------------------------------------------------------------------------------------------------------------------------------------------------------------------------------------------------------------------------------------------------------------------------------------------------------------------------------------------------------------------------------------------------------------------------------------------------------------------------------------------------------------------------------------------------------------------------------------------------------------------------------------------------------------------------------------------------------------------------------------------------------------------------------------taactaaatataaaattttttttaaaattcaaaataaaattgctcaatttttacagttaaaagaaagatcatgtttgccgaaaaaaactgacaaaaatgtgcttgaaaatttaatttaacttttaaatatttaaatgctacttatagttaaaataagtttattgcttttttttcttactgtnnnnnnnnnnnnnnnnnnnnnnnn---------------------------------------------nnaaaaatacaagtttctaaactttttgctgttattcaataattttgctgtaatttataaaaacaaaatgtcgctttggaagtctttaaaccgtttatgtttacattacaataattttagtaagagaattgtcacaaaaaatattttcaataatgggtaatttaaatactatgaattttatttagtaaataattcatcatacttcacttacttttcgagatttattagtttgcgcattagataaaagtaaattaaactaaaaaaaattttacattttagatgtcgtactcctatcattcatccgacgatttcaaaccgtcctgttcatatttctagtataaataatgatataatggaattttttgatgataaagaaaattggggagctagagaagtttcagttggtagatcatggaaagtagaggaacttagaattaaatcaaattctgatttacataaattatggtacatactattaaaagaaaaaaatatgttacttaccatggaacatgaatataatgtaaatactgaattgtaatttatatgtttagtattattaaattaaatgttggtacaagttcttttttcttgtatagagattgtataaagcaatgccaaatcctgaaagaattgataaagttgaagaaagtatggaaaatttagaaaaagttgtcagagaaagaaatcgagcatattatgaacttgaaactggaaaaacaggagaacgccctttctgtgataaaacaaattgttttggtataatttaaaaaaaatttatttaataaaaatattaatgataattatattttactattgtaatacaaatttttaaataatgttgtaggattaacttacaagtacaaaatgaaccagtataaagtaccaccttatttaaataaaaagtatttggaaaaagataagttagaaaatcaaacagatgatcatgtgtatattcaggactttttaacaagactcaaggaaaaaaaagctaaaagagatcattatcacagaaggtatgataattataactaattttttccataaaccattagtttctatattttataaactgaaattactgtatatattttaaaagtgtcttgggtataaatttgttcaatgctaaaatgaaattttttaaaaaaagtattactgtgctcttctttatacttaagtaattttaacttaaatataataatgaaaatgattattgctgtcagcaagcatattttttctgtttttatatcaatataataataattacattaattgattttgtggtacataaagatatatgtacatatgtatttaaaatttaatattaatttcagaggaaggtatatggttgtttgtaacttgcttaaacggtttcctgatttggatgaagaagcattaaaagagcgatttccagatgtgaatattgaagcagctaaaaaagataaaaattcaagaggcaatcgcgaaaatccttattaaagatatttaattttaggaaattaataaaattttttaaaaagc--------------------------acannnnnnnnnnnnnnnnnnnnnnnnnnnnnnnnnnntttataatagaaaatcttaaattcaggaataattcattacatttttggtaatagcaaaatagtcgtaaaaagcttattttgctaccaatcaaaaaccaaatggaagataatttttgcttaaattaacagtagtatttgtaccatgaaaaaatggcggatgaaagtgagagttatccat-----------------------------------------------------------------------------------------------------------------------------------------------------------------------------------------------------------------------------------------------------------------------------------------------------------------------------------------------------------------------------------------------------------------------------------------------------------------------------------------------------------------------------------------------------------------------------------------------------------------------------------------------------------------------------------------------------------------------------------------------------------------------------------

>EOG5B01FR_Protein_query

--------------------------------------------------------------------------------------------------------------------------------------------------------------------------------------------------------------------------------------------------------------------------------------------------------------------------------------------------------------------------------------------------------------------------------------------------------------------------------------------------------------------------------------------------------------------------------------------------------------------------------------------------------------------------------------------------------------------------------------------------------------------------------------------------------------------------------------------------------------------------------------------------------------------------------------------------------------------------------------------------------------------------------------------------------------------------------------------------------------------------------------------------------------------------------------------------------------------------------------------------------------------------------------------------------------------------------------------------------------------------------------------------------------------------------------------------------------------------------------------------------------------------------------------------------------------------------------------------------------------------------------------------------------------------------------------------------------------------------------------------------------------------------------------------------------------------------------------------------------------------------------------------------------------------------------------------------------------------------------GTTTGGTCGGACGATTGGTTAAAAACAATCCCACGGCAACTTCTGACGATTTTGAAAAAGCGTTCTTCGAAAATATTAAATGTGGTGAAGTTAAAGAAGAAAAAAATGATGATGTCGAAGCCGGTTGCTCCGAGGATTCTTTTTTCGAATCCAACATTAAAATGTGCTGGTGCCAAACGACGCAAAGTTTAAAATTCGTTTTTCAATGTAAAATATCTTCTCCTCAAATACTCGTGACTCTTAAACCCGAACAACATTTAAGAATATTGATCAGAGTTAAAAAAAAACTTCATTTGTACTGTTTCAAATTAGAGAAAAAAGTTTTGTGGCCGTGCATCGTTACTGTGAACACGGATACGGGAAGAGTAGAATTAGAATTTGCTAAAAAAGAAGAAGGTATTTGGAATTCGTACGGTATCGACGACAAAAATCACGGCATGATGATGTCTCGCGTCGACGAAATCGAAGCCTACCACAAAGCCAAAATAATCAGTATAAATCAAGTCACTCATAACGTGAGGACATTCGTTTTTAAATTTGTCGACAAGGTGCTCATGTGGGTTCCGATCGGGCACGACGTGAGAATCAGGGGGATAATCGAAGGTATAGACTACGCAAAACAATACACGCCCATTCCGCCGTATCTTCCTCACGGGGAACCCACGTTAAGGCACTGGCATCACGATTATATTTGCTTTATGGTAAAGTATTATAGTGAAGGCGCTCTCACGCCGTATTTGTTTGGAAAAAAAGAGCAAGACGTTGTGGAAATCGGAGGGATGAGCGGGAGTTTTAACATAAGAAAATTGAATAACGTTAAAAAATTATATCTGATAGCTGCTGGTTCTGGATTGTCACCGATGCTTCGAATCATAGTCTGGGCAATATCTAAGAAAGAACAAATGTGAGATTCACAATTAAGTTATCATCAAATGCAATAATTGTTTTCTTTTTTTTTTTTTTTTTTTTTTAATAAGAAAAATTACGATCGTTATTTTTTATTTTTAGAAAATCCATGAATCTCTTGTTTTTGAATCGATACGAAGAAGACATTATTTGGAAAAAAGAATTGGATCTTTTGTCGAAAAAAGAAAAATGGTAAGAGAGAGAGAGAGAGAGAGAGAGAGAGAGAGAGTGCGCCTACATAACACGATTATCTTTTGCGAAACCTTTTTTTTTTTTTTAATAATGCATATATCGTTTGTACATACGTATGTAGTGTTGTATCATTTATGCGTAGGTTTAAAATCACGTATTGCCTTTCTCAACCTAATTCTTCGTGGAACGGACTAAAAGGTAGAATAGATGAAACTATTCTTGATAATTATTTACTTATCAATAAGGACGAGTCCTCCAAGGATTCTCTTCTTAATGATTTTTTCATCTCTGCTTGTGGACCGATTAAATTTACTGAAATGATACAATCGTATGTAAAAATTACTAATAAATAAGAAAATTTTATGAAATTATATATTCGTTAAATTAATAAATAAATCATTTTTTTTTTTATTTATTTACAGATACCTGATTAAAAGAGGTTTCGAAAATTGCAGTCATTGTTTTATCGGATAATAAAAATAATT-------------------------------------------------------AGCTAATATTATAACGAAAAAAAA-------------------------------------------AAAAATTGTC----------------------TTTCTTCTTCTGC------------------------------------------------------------------------------------GCATAAGTAAAAG-------------------------------------

>EOG5B01FR_DNA_query

taagaatataataattattattattattattattaacaaagcgaaaatcaatttgaaattaatacatcattaataattctataataaattatttttatcgaaataatttttcttgtgaaataaattgactaaaatgtcaccgtcgaatgattaaattatatttttatttaaaaaacaaaattaataaaagtaattttaagaaattttatttttatcttgaattaaacttattaaaaaaaacaaagcttcagaaataaattttttttttaaccaaacagcattacgtgataactttattaaagtgattaattacttacctgctcattgcagaaagccaaaacctccatttttaaaatgctacgaagcaaatgataaagtaaatgttaagctctggtaccaaagttcacttgaatcaaatattaaacgcacacgattatattctaacgagtaggctgtccaaaagttttctttgttgtttccaatgtttgttttgacgcattgtcaaaaaaaaaaaaaaaaccttcattcgtgcactttgattttaattaccaaaggaccttaatgaacagaaaatttgccaattattgttattattattattatttttttttttttttatacaaataattttttaaaatcgtgaatgaaaaaaaataaaataaaaacaagttgtttatcgttattattattaattaaattaattcaattaatatcttatgcccttttttgatttttaactaatgaactaataaaatttaatatatatttccagggaatcccaggaataaggttgcccttaatcctggacactcgctcatggattggatcagattgggaaattcgggtaaagatttgaccggcgttggcggaatattaaaagatgtacctttagatgaattggcagaacataacagtgaagaaaacgcgtggataagtttaagaggtttgcttaaattttactgctgtcattaaattattattgattgaacaattaaggatgaattttaaattcatctgtaggaaaagtttacaatgtatcaccttacatgaattttcatcctggaggcgttccagagttaatgaaaggagttggtaaagatgccacaaagctattcacagaggtaggaggtattgtcttattccatactaagatttgaaaacttaatttttctcacataaataatatgtttgtccatgtgcatgttggaaactgtaaataatacaaaaaacatttttttttttattattggcaaaaaaattccttaataattttattaaattaaacgcgtgtttaacttacttttccatacacgtgcatatttcttcatcatcatcattcatcattcatcattcggattttgttcaaatattaattttgaaaaaataaaaaaaaaataaatcaacttttttttccgatttgtttttattgtattaaaaattaaaataaaatatccatatattatatttaaatacttatctcaggtatgattataaaacacatgcacgaaaaattaatgtttttttttttttttttggtgggggtgaccaattttccatgattaaagtaaatattgtgaaatgtagaatttgttgacttatttgactgtttgctcggaaactcactcgtaagtagatcgtgaaaaattattatttgcgtaacaacaatacacacaacagaaaatttttttaatttttaactatatggtgtgtctaaannnnnnnnnnnnnnngagagagagagagacagagagagagagagaaatttcgattttgattcaatttaaatgcaagaaaaaaataaaaataataataataataataatattttaaatccgttttataacgtacaataagaaaattttattttcagattcatccttgggttaattacgaatctattttgcaaaaatgtttggtcggacgattggttaaaaacaatcccacggcaacttctgacgattttgaaaaagcgttcttcgaaaatattaaatgtggtgaagttaaagaagaaaaaaatgatgatgtcgaagccggttgctccgaggattcttttttcgaatccaacattaaaatgtgctggtgccaaacgacgcaaagtttaaaattcgtttttcaatgtaaaatatcttctcctcaaatactcgtgactcttaaacccgaacaacatttaagaatattgatcagagttaaaaaaaaacttcatttgtactgtttcaaattagagaaaaaagttttgtggccgtgcatcgttactgtgaacacggatacgggaagagtagaattagaatttgctaaaaaagaagaaggtatttggaattcgtacggtatcgacgacaaaaatcacggcatgatgatgtctcgcgtcgacgaaatcgaagcctaccacaaagccaaaataatcagtataaatcaagtcactcataacgtgaggacattcgtttttaaatttgtcgacaaggtgctcatgtgggttccgatcgggcacgacgtgagaatcagggggataatcgaaggtatagactacgcaaaacaatacacgcccattccgccgtatcttcctcacggggaacccacgttaaggcactggcatcacgattatatttgctttatggtaaagtattatagtgaaggcgctctcacgccgtatttgtttggaaaaaaagagcaagacgttgtggaaatcggagggatgagcgggagttttaacataagaaaattgaataacgttaaaaaattatatctgatagctgctggttctggattgtcaccgatgcttcgaatcatagtctgggcaatatctaagaaagaacaaatgtgagattcacaattaagttatcatcaaatgcaataattgttttcttttttatttttttttttttttaataagaaaaattacgatcgttattttttatttttagaaaatccatgaatctcttgtttttgaatcgatacgaagaagacattatttggaaaaaagaattggatcttttgtcgaaaaaagaaaaatggnnnnnnnnnn----------------------------------------------------nnnnnnnnnntttttttttttttaataatgcatatatcgtttgtacatacgtatgtagtgttgtatcatttatgcgtaggtttaaaatcacgtattgcctttctcaacctaattcttcgtggaacggactaaaaggtagaatagatgaaactattcttgataattatttacttatcaataaggacgagtcctccaaggattctcttcttaatgattttttcatctctgcttgtggaccgattaaatttactgaaatgatacaatcgtatgtaaaaattactaataaataagaaaattttatgaaattatatattcgttaaattaataaataaatcatttttttttttatttatttacagatacctgattaaaagaggtttcgaaaattgcagtcattgttttatcggataataaaaataannnnnnnnnnnnnnnnnnnnnnntttttttttttttttttttttttttattttggggagccaataattcaaccgtaatcagcatttgttttttatttattattttcttatatttattagcgattgaaaattttcctcgttttatattattttcatttttctttttcttctgaaaatttatttaataaattttattgttattattattaatattattattattattattattatttgttttcctattaattattatgtacgtaaaaacaaattttttctctctctccatgtttttaaactttta

>EOG5B2WNF_Protein_query

ATAATTAAAATGTTAGATAAAAAGTGAATTAACGAAAAATCAATCACCCTGATATTTAACCTAACTCTCAAATATATTAATTATTTTATATATTAAACGGTAATAAATTTATAAATGTTAAAAATCAATTTATTTACCCCTAAATTCGTAAAAGTCATGATGGCTTTTTACAAATGGCTAACTTATTGAATCGTGGCAGTAGATTTCTTGTAAATGACTCTTTAAATTACGAGCCTACATTTAAAGTTGTCACGAGGTATTTTTTTAAAAATAATTAATTAACTACTTTTAATGATTTACTTTTGTTCACTTATTTTCAATTTCAATATTTAAATCAATTGGTATCATGTATAATTAAATAAATAAAATCTATTCAAATTCGTTTGTAGTACTTTTACCATATAAAGATGAAAAATCATTTGAAAGTATTGAATTTAATACAAAAAAAAAAAAAAAATCCGCATCAACTTTGTTTATGACATATATAAAAATAAAAATTTAAAATAAATAATACATTATAATAAACAAAAAATTTATTTTATTTTAATTAAAAAAAAAAAATGTAAATATATTTGATTCGGTAACGAGAAATTTTTACGTAAGACAATCATTTAATTGACAATCTCACATTAGTTCACAACTTAAACTTTGACTTTTTTTTTTTTGCATACTCAAATACAACTCTTGATTCTGTAAAAATTAAAAAAAATTAATTAAAACTCATTTTAATTATTATTTTTAATTAAATTTAACGCACGCGAAATCAATTGAAAATGTTGTTTCCAAAGCAACACCTTATCGGACAGCCATTTATTTATTTACTTTATTATTATTATTATTGTTGTTATTATTATTATTATAATTAGTATGAAAATAAAAAAAGTGTAATTTTACGTTTTTTTTTTTTTTTTTAAAAACCATATTGTCTGTTTTAAAAATATGAAGTTGAAAAAGTTTCTACTAAGGTATTATCCACCAGGTGTGATTTTTCTTTTTAATAATTTAAAATTTAAAAATTATATCGAATTACAGTTAAAAACAAAAAACAAAAATACAATTTGATAATTTTTAATTTATGACATAAGTGATTGATAATAATTTTAAATTAAATTTCAAAGCGTTTATAGCGTTTACGATTTTTTCAGGTATCGGTCTCGAATACGAGCAAAATCAAGAAACCAAAACCAAAATGATCGATCTATTAGACTTGACGTTGAAGTGAGTTTTACATTTAAAAAAATAAATATTCCGTTTTGACTGTGAAATTACATCATTTCTCTGTCGTTTCAGAACAGATGTCCGTGAGCTATCCGATAAAATTGCTAAAAATGAACCGTTGATAACGCAAAACATAGTCGGCCAACTTGGAAACACGATGGAGAAAATTCAAAACAAACTTCGTGAATCTGCCAATAAAAGATTTCAAAATTATTTAACGTTGAAAGCTCATTTACTACCTGTGACGAATGTTGCGTTTGATAAAAACGGACAAAAGTGACATTTTTTTTTTTTTTTGTTGCTCACAACATTCGATTCATTTCTTTTCGTAATATTTTCAATTTAATTTAATTATTCAGGTGCATTACAGGAAGCTACGACAGAAAGTGCAAAGTCTGGAATGTTGAAGATGGAAAAGAACTTTTAACACTGGAGGGACACAAAAATGCCGTCTATTCAGTCGCTTTTAACATGCCCACGTGGTAAGCTAAAATATACAAAGTTTTTTTTTTTTTAAATGAAACTTATTTTTGTTTAATAACTATTATTTTTTTTCTCTCTCTCTCTTGCAGTAATCGAATATTAACGGGATCCTTCGACAGAACTGCCAAATTATGGTGCTCTCAAACGGGTAATTGCTTGAGAACATTATGGGGTCACACGGCAGAAGTGAGTTTTTTTTTTTTTTTTTTTAAATACAAATACTACAACATAATGTTATTCAATATGTTAATGTTTTCTCTAATAGTACACTGAAATGGAAAAATGTTTGTTTTTAAGGTCGTGACGGCTAAATTTGACAGTAAGCAACAGCTCATTGCGACGGGTTCCATGGACAGAACGGCAAAATTATTTGACGTATTAACAGGTAGCGAGGACGACTTATTAATTGTAAAATTATTTTTCGATAGACAATGTTATTCTTTTTTTTAGGCCAAGAAACGGGAACTCTTCGAGGCCACACAGGAGAAATAACTACTGTAGATTTTGATAAAACTGGAAATCAATTGATAACGAGCTCCTTCGACAGTACCGTCAGCGTGTGGGATACTAGAACGAATAAGTAAAAATATACCTTGTGAATATAATGTTTGACATTTTTTAAAAGAAGCCAATAAAAAAAAATCTGATGATTTCAGGCGAGTCGGAGTATTGATAGGTCACAGAAATGAAATTTCAAACGCGTTGTTTAATTTCGATTGCAATTTAATAGCGAGCAGTTCTGTTGATAAAACATCCAAAGTTTGGGATCCTCGTATGTTTACCTGTCTTGCTACAATCACGGGCCACGACAGCGATGTAAAATCATTATTTTCAATTTATCTTGTTTTTAATATATTATAATTTTTAACTTTTTCGATAAATTTAACGTACCATACCTCTTATCAGGTGTTAGATATAGCTTTCGATTGTTACGGTAAGAGATTGGCGACGGCCAGCACCGATTGTACCGCTCGCGTTTGGGATGTATCCTCCAATTTTAAATTATTATCGACAATGGAAGGTCATAAGGATGAAGTGTCACAAGTTTGCTTCAATCCTGGTGGAACTCAATTACTGACAGCTTCTAACGACAAAACGGCCAGAATTTGGAATCCGGACACGGGATGTTGCATTCAGGTAATATTAGTAATTATGCAGTTACAAATAAAAAATGAATTTTTTCTTTAACAGCAACCTGAAAAAAACTTTTCTTTTTTTTTCTTCATTTTACCAACTACCAGGTACTGCAAGGACACTCTGATGAAGTTTTTTCAGGTGTTTATAGTAATAATGGTGACGTTTTAATAACAGCATCAAAAGATAATACTTGTCAGATCTGGAAATAAAAAAAAAGAATTAATTTTTTTTAAATATCAAACGACATATCTTTTGTAAGAAAATGATATGGGAAAAAAAGAAGGTGCTTGATGTAATAAGTGATTTTTCTAAAAAGCTTTCAAAATGTGTGTGTGTTTGGAGATAACCTCACTTATATGCATTTTTTTTTTTTAAAATCCTAATAAAAAAAAAAGAAAAAAATTTTGAGAAGTTACCTTAGATTGTTTCAAGTTTTTCATTGTGATTCCAACAATTTAATGTTTTGTTGTCACAAATGGATATTATCAATTCCTGATGGCCTGAATTTAACCATATGTGATCTGTAACTGTTGGTAAAAATTCATTACCAGGACTATGACCATTATGAGTAAAAATATTTTTGTAATTATTATTAGCAATTCTAAAAACTTTAACAAATGCATTTAAACCAGATATAGAAAATTTGTCTTCTTTTTCACAAAATTTCAATTTTAAATTATCAATGGATTTTCTATGAGTATGAATGCTTCCTTGATTTTGAAATAGTAAAGAATTTGTTCTTTTATCAAATATGCAAAAATTTTCAGAGTTTGATAGAATTCCAAAATAATTAGGGAAGGAATTCTTATCATCATTTTTAGACATTGTCCAATTTTCAACAATATTTGGTTGCTTTGTTTCATTTACAATATTTATAAAACATTTTTCAGTTCTTAAATCATAAATTGACAAATCACCAGTTTTTGAATTTAATATTGAAATTGTATTTTCAGTCAATGGCAGGCATTTAATATCACAATCATCATGACAGCTGACTCCACTATCTTCAAGATATTGTTGAGTAAAATTATGTTGCTTGCATTTAAAATGATAATCAACTTCCTGAATTTTTTTTTTAACATTTAAATCAATTAAAACAATTGGATGATGATTTGTTTTACAGCAAACTATGTTATTGGTTTTATTGATAACATATAATTCTGAACTCATCCACGGCAAAAAAGAAATACTCTCTGGCAGTGTTTTTCCTAAAATAAAAATTTATTTTTTATTTTCTAATTTTTAAACAGATTTTTTCAATAATTTAAGACTTACCTTGTCCATTATTATAAAGACTATAAATTTTTAAACCGATTGTGTTTTTCTCAGAAACAATTACTTTTTCTTCATTTGGTAATCCATCCATTCTATGCACTAAATTTTCTGTATACACTCCTGATAAAACTTTGAAATCATTATCTTTACTTCTACCTTCTTCTTCGGGAGGCAATGCAAGTTTTTTAGGTAACTGTAATCGCAATAATTCTGAATTTGTGGCTTTTTCATTTTGACTAGCAATGCAAACTGATAATTTGTCCATTTGAATTAAATGGTTTGGAACATTATTTAAATTAAACATTTGCAATGTGTTATATCTGAAATGTAATTTTATTAAAAAACTGCCTGATAAAAAAAAATTTGGTGTTTCATATA

>EOG5B2WNF_DNA_query

------------------------------------------------------------------------------------------------------------------------------------------------------------------------------------------------------------------------------------------------------------------------------------------------------------------------------------------------------------------------------------------------------------------------------------------------------------------------------------------------------------------------------------------------------------------------------------------------------------------------------------------------------------------------------------------------------------------------------------------------------------------------------------------------------------------------------------------------------------------------------------------------------------------------------------------------aaaaaccatattgtctgttttaaaaatatgaagttgaaaaagtttctactaaggtattatccaccaggtgtgatttttctttttaataatttaaaatttaaaaattatatcgaattacagttaaaaacaaaaaacaaaaatacaatttgataatttttaatttatgacataagtgattgataataattttaaattaaatttcaaagcgtttatagcgtttacgattttttcaggtatcggtctcgaatacgagcaaaatcaagaaaccaaaaccaaaatgatcgatctattagacttgacgttgaagtgagttttacatttaaaaaaataaatattccgttttgactgtgaaattacatcatttctctgtcgtttcagaacagatgtccgtgagctatccgataaaattgctaaaaatgaaccgttgataacgcaaaacatagtcggccaacttggaaacacgatggagaaaattcaaaacaaacttcgtgaatctgccaataaaagatttcaaaattatttaacgttgaaagctcatttactacctgtgacgaatgttgcgtttgataaaaacggacaaaagtgacatttttttttttttttgttgctcacaacattcgattcatttcttttcgtaatattttcaatttaatttaattattcaggtgcattacaggaagctacgacagaaagtgcaaagtctggaatgttgaagatggaaaagaacttttaacactggagggacacaaaaatgccgtctattcagtcgcttttaacatgcccacgtggtaagctaaaatatacaaagtttttttttttttaaatgaaacttatttttgtttaataactattattttttttctctctctctcttgcagtaatcgaatattaacgggatccttcgacagaactgccaaattatggtgctctcaaacgggtaattgcttgagaacattatggggtcacacggcagaagtgagtttttttttttttttttttaaatacaaatactaaaacataatgttattcaatatgttaatgttttctctaatagtacactgaaatggaaaaatgtttgtttttaaggtcgtgacggctaaatttgacagtaagcaacagctcattgcgacgggttccatggacagaacggcaaaattatttgacgtattaacaggtagccaggacgacttattaattgtaaaattatttttcgatagacaatgttattcttttttttaggccaagaaacgggaactcttcgaggccacacaggagaaataactactgtagattttgataaaactggaaatcaattgataacgagctccttcgacagtaccgtcagcgtgtgggatactagaacgaataagtaaaaatataccttgtgaatataatgtttgacattttttaaaagaagccaataaaaaaaaatctgatgatttcaggcgagtcggagtattgataggtcacagaaatgaaatttcaaacgcgttgtttaatttcgattgcaatttaatagcgagcagttctgttgataaaacatccaaagtttgggatcctcgtatgtttacctgtcttgctacaatcacgggccacgacagcgatgtaaaatcattattttcaatttatcttgtttttaatatattataatttttaactttttcgataaatttaacgtaccatacctcttatcaggtgttagatatagctttcgattgttacggtaagagattggcgacggccagcaccgattgtaccgctcgcgtttgggatgtatcctccaattttaaattattatcgacaatggaaggtcataaggatgaagtgtcacaagtttgcttcaatcctggtggaactcaattactgacagcttctaacgacaaaacggccagaatttggaatccggacacgggatgttgcattcaggtaatattagtaattatgcagttacaaataaaaaatgaattttttctttaacagcaacctgaaaaaaacttttctttttttttcttcattttaccaactaccaggtactgcaaggacactctgatgaagttttttcaggtgtttatagtaataatggtgacgttttaataacagcatcaaaagataatacttgtcagatctggaaataaaaaaaaagaattaattttttttaaatatcaaacgacatatcttttgtaagaaaatgatatgggaaaaaaagaannnnnnnnnnn---------------------------------nnnnnnnnnnnnnnnnnnnnnnnnacttatatgcattttttttttttaaaatcctaataaaaaaaaaagaaaaaaattttgagaagttaccttagattgtttcaagtttttcattgtgattccaacaatttaatgttt-------------------------------------------------------------------------------------------------------------------------------------------------------------------------------------------------------------------------------------------------------------------------------------------------------------------------------------------------------------------------------------------------------------------------------------------------------------------------------------------------------------------------------------------------------------------------------------------------------------------------------------------------------------------------------------------------------------------------------------------------------------------------------------------------------------------------------------------------------------------------------------------------------------------------------------------------------------------------------------------------------------------------------------------------------------------------------------------------------------------------------------------------------------------------------------------------------------------------------------

>EOG5B2WP1_Protein_Query

CTAACATTTTTATTTTTATCAAAGCGAAAATTATCTTTAAATATATATACTTTTCACAAGAACACAAAATTTTAATAAACTTTACCAAATAAACAATAAATAAGTCTTGCCGCTCTTTTTAATCTCATTTTTATTTTGTGATAAATCACTTTTAGACTATAAAATTTTACATTTCATTTTATTTCTAATAATATTGTAAAAAGTTTTTCCTTGCGTAGCCATCTTTGTTGACAGAATGACATTTGCTATTATTAAGTGGCGCGTCAAATGAAAGGAAACAAGTCGAAAAAATTAAATTCGGTCGTTTTAATTTACGGCAAATATTCGCTTACATTTACTACCTCACAGGTTTCGTTCATTTGCTTAAGTATTTTTTTTAATTAATTTTTTTTTTTTTTTCAAAGATTGAAAAAATTATTTTTCAATAATTTTATGGAGCGAGTATTATAGTCGTACTAAAATAATAATATGACATCCTATTCGTCTCATTCCGTGAGTTTTATTTTTAAAAAAAATTTTTTTTTTTTATTTTAACTCAAATGATTGTATTTGTGTTCTGATCACTTGTAAAATTTGAAGTATTTAATTTATTTTTATATTATTTTTATGTTTCAAACATTTTATTATTTATATATATTTAAATAATTATTTTAAATATTTTTTTGTGTTTGGCCTCTGATAAATGATTTCAAATAATATTGCATTTTAACCACAATTGTTTACCTCCGTTCTAGGCTACTGCAACGTCAGGTGGAGTGAAACCAGTTGGATCTGTTCAAGAATATTCAATAAGAGTACCCAAGTAAGTAACATACATTATACTACCAGAACTATGTCTGATTCAATTAAGTTTGCTAATTAATTGTTTTTAATTATTTATTTATTTATTTTTCCTAACATACTAATGCTCTGAAACAACTGACCTACTTTGCTTTTTTAGAAATACAAGAAAAAAGTACCATGTTATGAGATTTAACTCTAGCCTTTCCGTTGACTTTGTAAAATGGAAGACTCAAAACATTCAAGTCAAAATGGAAAGAGAAAATAATTTTAAAGAATATAAGGGTGCTGAAGAAGAAATACCCAAGTAAGTAATTTTATTAAAATTTTGTTAATTATGAATTCTTTCACAATTCTTACTTTATAAGATTCGATTTAAAGAATTTTTTCTATAATTACTTATCGTAATACTTTTTAACTTATCGCAGAAAGTTTTTTTGTCTTAAAAATTGTTTTTTTTTTTTTTAACATAACAAAAAAAAAATTTTAGCATTTCGAATTTTTTTTTAAATGACTAAATTTTCATAGTTTTATATCTTTTTTTTGATGTGTGTCAAATTTTATTGTTGATAGTAAAATATTTAGACATCCAAAATTTTTTTTTTTTTTTTTTTTCTGAAAAAGTTTGGCAAATTTTTTTTCTTCAGTAGTTTCAGAGGCACATGCAAACATCTGATTTAAATTTCTAGATTTGGAGCAGGTAGTGAATTTGGTAGAGAAGCAAGGGAAGAAGCTAGACGTAAAAAATATGGTATAGTAGCAAAAAAGTATAAGCCAGAAGACCAGCCATGGATTTTAAAGCAGGGTGGAAAAAATGGTAAAAAGTAAGCATTATAGCAAGAAAATGATCCATAGTAATTTTTTTTTTTTTATATATATATTTTTAGTCCTGAATAAATTAAGTTCGCTAAACAATTTTTGCAACTGAAATATTAACATTAATTTATTTTTTTAGATTTAAGGGTATTAGGGAAGGAGGAGTTGCTGATAATACTGCTTACTATGTTTTTACACAAGCCGGAAATGAAATAATAGAAGCTTATCCATTACAAGAATGGTATAAATTTCAACCGATTCAAAGATATAAAGCTCTGACTGCAGAAGAAGCTGAAATCGAATTCGGAAGGTGAACTTAGAATTAAATTAATATTTATGAGAAGTTATATAATATCATAATTTTACGTGAAGTATATTGCGGTCATTAATGTTTTTCTTTTCCTTTCTGTTTTTTTTTTTTTTTTTAGACGAAACAAAGTAATGAATTTGTTTTCGGTCATGATTAAAAACAGATTCAAAAATGAGGAAGACGAAGAATTAGAAGATCCGGACAAACCAAAAGGGAAAAAAGGTGGTGGAAAAAAAAAAGAAAGAGGTAACGATTTTTCACAAATAATTACAGTATCGTAGTTGAAAAAAAAAAAAAAAAAAAAAAGGAAAAAGAACACTAACAACAGAAAAATTTG-TTTTTTTTTTTTTTCTTTTTACAGATTTACAAATCTCTGAAATGGACGAATGGATAAATTCCGATGATGATTTATCAGATTCGGACGACGATAAGGGCAGCGGTGATGATGACGATTCTAAAAAAAATAAAAAGAAAGGTAATAAAAAAATTGCTCAAAAAAAAAAGAAAAAAGATTCTGATGATGAAGCCGTGGAAGAAAGCGACGACGGTGACGAGGAAGGAAGAGAATTAGACTACATATCCGATTCGTCTGAAAGGTATTTTTAAAAAAAAAAAGAAATTTTTTTACTATTTGTTGTCTCATGCTAATCGAATGCATGTTGAAAATATTATGCAAATTACAATCGATTCAAATAAATAAACTAACAAATTTATTATTTATTAGTGCATCCGACCACGAAATGAAAGCCAATAAAGAATTAAAAGGAGTAGCAGAAGAAGATGCGCTTAGAAAATTGTTGAATTCAGAATCAGAAGATGAAGAGAATGAAGAAAAGAAAGAAGAGGTTAGAGTTTTTTTTTTTTTTTTTTTAAATAATAATAATAATAATTATCTTTTTACGCATTCATTAAATTTTGATAAAACCATTCAAGTGCTTTTGTATTTTGTTCAAAGTAATAAAAGAGTATATTTTTCTCTTATGTTGAATCGTGTAATGGAATTTAGGAAGCGTCTCACAGCGAAGATGAAGAATCTAAGAATAAAAAAGAAAAAGATGAAAAAGCAAACGGTGATAAATCAAAAGAAGATGCTAATAAAAAGAAAAAACCATCAAAGGACGCTAAACCCAAAAATAAAGGCCTTAAAAAAATCGGAAAGAACGAAGACGATTCTTCTAGCGAGCTCAGTCCAAATAGCTCTGATAGTGATAGTGATGCGGGAAACAACAAGTCCAAAAGCGGAGTAAAATCAAAGGATTTGAATAATAGGTAAATTAAAAAGAAAATAATTACACAAAACCAAAAATTTAAATTAAAAAAAAAAAAAAAAAAAAAACTAAGTTCCGTGTAAATTTTTTAAAATTTTAGCGGTAATAATTCGGGATCTGGTTCGACTCCGACGTCAAATAGCGGACCGAATACCGGAGATAAAAGGAAATTAACAAATAGCGAATTAGCCGTGGCAGCAGCTAAAAAAGCTAAATTAGATAATTCGTCCATATCGAGCGGTTCAAGTCTATCGTATTTGAATAACAGCAAGTGAGTAATTTTACGGATCAATGAATAAGCTCAGGACGATTCGAAATCAAAAATAATAATAATAATTCGATACGTAAATTAATGAAACAACAATGTTAATATTAATTGTTGATAATTTTTTTTTTTTTTTTTTCTAGCGAAACGGGAGTGACGGAAGAAGCCGTGCGTCGTTATCTAATGAGAAAACCGATGACAACCATAGAACTATTACAAAAATTCAAAAGTAAAAAAACAGGTTTGTCATCGGATCAACTCGTGAACGTAATGACGCAAATATTAAAGAACATAAATCCGGATAAGCAAATGATAAAGAAAAAAATGTATCTCTCCATCAAGCCCACGTAATAATAACGCGAAACGACGACAAAATGAAAATACGGGAAAAAGT-------------------------AAAATTACAAAAAAAAAAAAAAAAAAAATT-----------

>EOG5B2WP1_DNA_query

-----------------------------------------------------------------------------------------------------------------------------------------------------------------------------------------------------------------------------------------------------------------------------------------------------------------------------------------------------------------------------------------------------------------------------attatttttcaataattttatggagcgagtattatagtcgtactaaaataataatatgacatcctattcgtctcattccgtgagttttatttttaaaaaaaattttttttttttattttaactcaaatgattgtatttgtgttctgatnnnnnn---------------------------------------------nnnnnnnnnnnnnnnnnnnatatatttaaataattattttaaatatttttttgtgtttggcctctgataaatgatttcaaataatattgcattttaaccacaattgtttacctccgttctaggctactgcaacgtcaggtggagtgaaaccagttggatctgttcaagaatattcaataagagtacccaagtaagtaacatacattatactaccagaactatgtctgattcaattaagtttgctaattaattgtttttaattatttatttatttatttttcctaacatactaatgctctgaaacaactgacctactttgcttttttagaaatacaagaaaaaagtaccatgttatgagatttaactctagcctttccgttgactttgtaaaatggaagactcaaaacattcaagtcaaaatggaaagagaaaataattttaaagaatataagggtgctgaagaagaaatacccaagtaagtaattttattaaaattttgttaatnnnnnnnnnnnnnnnnnnnnnnnnnnnnnnnnnnnnnnnnnnnnnnnnnnnnnnnnnnnnnnnnnnnnnnnnt-------------------------------------------ttttttttttttttaacataacaaaaaaaaaattttagcatttcgaatttttttttaaatgactaaattttcatagttttatatcttttttttgatgtgtgtcaaattttattgttgatagtaaaatatttagacatccaaaa-----ttnnnnnnnnnntttctgaaaaagtttggcaaattttttttcttcagtagtttcagaggcacatgcaaacatctgatttaaatttctagatttggagcaggtagtgaatttggtagagaagcaagggaagaagctagacgtaaaaaatatggtatagtagcaaaaaagtataagccagaagaccagccatggattttaaagcagggtggaaaaaatggtaaaaagtaagcattatagcaagaaaatgatccatagtaatttttttttttttatatatatatttttagtcctgaataaattaagttcgctaaacaatttttgcaactgaaatattaacattaatttatttttttagatttaagggtattagggaaggaggagttgctgataatactgcttactatgtttttccacaagccggaaatgaaataatagaagcttatccattacaagaatggtataaatttcaaccgattcaaagatataaagctctgactgcagaagaagctgaaatcgaattcggaaggtgaacttagaattaaat--------------------------------------------tannnnnnnnnnnnnnnnnnnnnnnnnnnnnnnnnnntttttttttttttttttagacgaaacaaagtaatgaatttgttttcggtcatgattaaaaacagattcaaaaatgaggaagacgaagaattagaagatccggacaaaccaaaagggaaaaaaggtggtggaaaaaaaaaagaaagaggtnnn-------------------------nnnnnnnnaaaaaaaaaaaaaaaaaaaaaaggaaaaagaacactaacaacagaaaaatttgtttttttttttttttctttttacagatttacaaatctctgaaatggacgaatggataaattccgatgatgatttatcagattcggacgacgataagggcagcggtgatgatgacgattctaaaaaaaataaaaagaaaggtaataaaaaaattgctcaaaaaaaaaagaaaaaagattctgatgatgaagccgtggaagaaagcgacgacggtgacgaggaaggaagagaattagactacatatccgattcnnnnnnnnnn-----------------------------------nnnnnnnnnnnnnnnnnnnnnnnnnnnnnnnnnnnnntatgcaaattacaatcgattcaaataaataaactaacaaatttattatttattagtgcatccgaccacgaaatgaaagccaataaagaattaaaaggagtagcagaagaagatgcgcttagaaaattgttgaattcagaatcagaagatgaagagaatgnnnnnnnnnnnnnnn---------------------------------------------------------nnnnnnnnnnnnnnnnnnnnnnnnnnnnnnnnnnnnnnttgtattttgttcaaagtaataaaagagtatatttttctcttatgttgaatcgtgtaatggaatttaggaagcgtctcacagcgaagatgaagaatctaagaataaaaaagaaaaagatgaaaaagcaaacggtgataaatcaaaagaagatgctaataaaaagaaaaaaccatcaaaggacgctaaacccaaaaataaaggccttaaaaaaatcggannnnnnnnnnnnnnnnnnnnnnnnnnnnnnnnnnnnnnnnnnnnnnnnnnnnnnnnnnnnnnnnnnnnnnnnnnnnnnnnnnnnnnnnnnnnnnnnnnnnnnnnn-----------------------------------------------------------------------nnnnnnnnnnnnngtataaattttttaaaattttagcggtaataattcgggatctggttcgactccgacgtcaaatagcggaccgaataccggagataaaaggaaattaacaaatagcgaattagccgtggcagcagctaaaaaagctaaattagataattcgtccatatcgagcggttcaagtctatcgtatttgaataacagcaagtgagtaattttacggatcaatgaataagctcaggacgattcgaaatcaaaaataa-----------------nnnnnnnnnngaaacaacaatgttaatattaattgttgataa-tttttttttttttttttctagcgaaacgggagtgacggaagaagccgtgcgtcgttatctaatgagaaaaccgatgacaaccatagaactattacaaaaattcaaaagtaaaaaaacaggtttgtcatcggatcaactcgtgaacgtaatgacgcaaatattaaagaacataaatccggataagcaaatgataaagaaaaaaatgtatctctccatcaagcccacgtaataataacnnnnnnnnnnaaaaaaaaaaaaaaaaaaaaaaaaattgtcattcacatcgtgcacatcgtcataataataaaaataataataataaataccggaagtcggaa

>EOG5B2WPB_Protein_query

--------------------------------------------------------------------------------------------------------------------------------------------GGAAAATGTAAAACCTGTAAAAAATTTTTAAATGACATGATTTAAGCTTAAAAAAATTTAAATGGGCTAATACATCAAAAAAGTAAAATTAAGTAAAAATTAAAAACTGATTTTTATTATTGAATAATGAATTAATAAATAAAACAAAACTTAATAGGAGACAAATTGTCATTTAGTTAAAGGAACAATATCAAAAAAATGCATAAATTAAGATTTCCCAAAAGACAAATCAAACTTAAAACTAAAATATAATTTTGTGTAGTATATAGTATAGTAATACCAGTTGATGTAGTGGTAGAAACTGTAGTAATACTTGAAGTTACAGATGGATTAACTCCAGTTCCAAAAACTAAGTTTGAAGTAGTGGGTATAGCAGATCCTAGGTTTGCATTTGGCATTGTAGATGAAGCAGAGGTGACATTTGCTGAAGGAAACGTAAATCCAGTATTAGATGTTTGAGGAGTAGAAGATGCAGTTGTTCCAAACCCAGAAACATTTGTAGATCCAAAGGTTCCAAAGGTAGTCAAAGATGTATTTACTGATGGAACCCCAAATGTAAAACCAGTTGAGGTCACAGAAGTAGTTACTGGTGGTTTAACTCCAAAACTGGTATTGAAAGCAGGAGCTCCAGGTTGAGTTCCCATTAAGTTTGAAACTCCTGCAGTTCCAAATGATGTTGCCGTGTTTGTAGTAGCAGCATTGGTAACAGTACTAGAAAATCCGGTAGAGGCTTGCTCCGGTGGTCTTCAAGCATTTCAAAAATTTTAATGAGATATATACAAAATTTATAAATTATTATTAACAAATGTATAGTACACAAAACGCTTTTTCAAAATTTTAAATACTCACTTAGAACCGAATAAATTTGTTCCCGATGAATTAAAACTAAAATTTCCAAACCCAGACATTTGATTGATTAAAACTTAATTTTAAAAACTATATTAGAATAATGGTATCAAGAACACTAATAAAAGTAATCTAACAAAATTAAATATAATTGCTTTAAAAAAAAATACAACGTTTTTTGTAATAAAAGGCAACTTAACTAATGTCTAGTTACACGTTGAAAGACATGTTTACGTTCAGTATTCACGAGACAGTTTTGTAGAAGGAAGTTTTAAAGAAAATATTTTATATTTTTAGTTGATATAGTATTTTTTTCGTCATTATTATTAATCTTTTATATTTTTAAAATGGATTTTCAAAATAGAGCTGGAGGAAAAACTGGTGGCGGAGGGGTAGCAACTTGGTCAGAATCTAATAGAGATCGAAGAGAACGGTTAAGACAATTGGCTTTAGAAACAATTGATATTAACAAAGATCCTTATTTTATGAAAAATCACTTAGGTTCTTATGAATGTAAACTGTGTTTAACTTTACACAATAATGAAGGCTCTTATTTGGCTCATACTCAAGGAAAAAAACATCAAGCTAATTTGGCTAGAAGAGCTGCTAAAGATGCTAAGGATGCACCTCAACAACCTGCGCCTGAAAAGCCAAGGGTAGATGTTAAAAAATTTGTAAAAATTGGTAGGCCAGGTTACAGAGTCACCAAGCAAAGAGATCCTGATTCTGGACAACAAAGTTTATTATTTCAGGTTCGTAATGATGTTGAATATAATACATATATTATTCATCTTGAATTCATTTAAAAATTACTTAAATTCTCTGTTTAAAATTAAAAAAAATTAATTAACATTTTGATTTATGAAATAAAAAATTTTTTTTTCCCATTTTTAAAAATTTAGTTATGATTAATGTGTATTTTTTTTTTTACAGATAGACTACCCAGAAATAGTTGAAGGTGTTTTACCACGCCACAGATTTATGTCTGCTTATGAACAGAGAGTTGAACCCCCTGACAGGAAATGGCAATATTTGTTGTTTGCAGCTGAGCCTTACGAAACAATTTCTTTTAAAGTAATTTTGTTTTTAATTAAAAATCTGTATTTATTAGGTTATTTTTTATTTTAAATTTAGTTTAAGTAATTTTTCATAATGAATCTACGTTTGGTGCTTTGCAACTTAGTTTTTTAAATTATAATTTGATTGCTTTTTTGCTATAATATGATGTTAATTTACAGAAAAAAAACTGTTACACCTTATTAAAATTGTTATTAAATTTTTTTCAGGTACCTAGTAGAGAAGTTGATAAATCAGATACAAAATTTTGGACTCACTGGAACAAAGATACCAAGCAGTTTTTTTTACAATTTTCATTCAAAATAGAACCCAAGCAACCTCCTAAACCACCAACAATTGGTAACCCTCCTCCTCCATTAATGAAAAATCAAATTTTGCCCCCTAACTTTGTTCCTCCCCCTCCCATGCCTCCTAGAAGTATGGTTCCTCCTCCTCCAAGATCAATAATGCCCTCAGTAGCTCCTCCTTCTATGTTTAATTTAATACCACCCCCTCCTCCAATAGTCAGTACCACATCAAGTAATTTACCAATACCACCACCTCCTCCTTCACCTCTCCTGAGAAATTAAACTAACTTTAAGTTAGTTTTTTTAATTTCATTTGTATCTACAATTTAAAGAAATGAAAATTTCCTACCAAAATTTTGCTTTATCTATTATCTTTACAATTTTATATTTAATGCATTAATTTAATGTTTAATGTAAATTTTGTGAAATCCCATTTTTTAAAATTGATAATTTTAAAAAATAATTAAAAATAAATTTAATTTGCTGTAAAATAATAATTTTTAAATACATTCTCACAAAAATAAAATGGACAAGAAATTTAATCAACATTTTAATGAACATGTGATAATTCTGCATTATGTGTAAAACAGTGAATAGTTTTTTTCTAAATATTTAATAGATTATGATAAAAAAAATAGTATTTTTTTTCTTTTTGCACACATTAAATAGCACTTAATAGTATAGGACAAATTAAAGACATTTTTAAATAATTAAAAAATTTATTTGATAGAAGAAATACAATTATGCTTGGAGAATACAGGCTCCCCCAACAGCTTTAATTTTGTCTTCTGCTCTCTTAGAAAAGAACTTTGCCTTGACAATGACTGGTTGTTTTGGTAATCTACCCTTGCCAAGAACTTTGTAATAGCCCTACGTAAAAAATTACAATTTATTATTATTTAGTATTAGCCTAGCTTTTTAATCAATTTTTTAAAATATTACTTACAGCTCTAACAACATCTATAACTGGAGCTTTTTTTTCTTTACTATTAGCACATCTTAATCTAGTGTCTGCACTTACTAGAGTCCAAATTTTATCTAAATTAATAGCTTTGCACCATCTAGTATTTCTGTTAAGATGATAATTTCTCATTCCCAACTAAAAAATTACAATTTGATTTATTACAAGAAATCAAAAAATTAAAAATTACTTGCTTGAATTATTACAAGTTTTTCACCTTTCCAAAATAACCAGGGTGGTATTTGTCAAAGTTAATTCTGTGGTGGTGCATACCTCCTGCATTACCTCTACCACCAGGATGTTTACGGTGTTTACCAATACGCCCGTGTCCATGTGAAACATGACCTCTTAATTTACGAGTCTTCTTCTTATTGGCAGCCTATTAATAATTATTAAAAGTATTAATATAATTTTTAAACCAAAAACATTAATAAAAAAACCAATGGCTTATGTTTAAAATCTAACATTTTTAATTACAGAAAAACCCTATTTATAAATACCATTCAACTTTGTTACAATGAAACAAAAAACATTTAATTCATAATTTTAATTCTACCTCCACTACGAATTAAAATAAAAAAGGATTGCTTTAATAAAAAAGAGTACAATTAAATAAATAAATTATTGGAACTCACCATGATAAAATTAAAACGTCAGGAACAAATGCAACAAGACGTTGGCGACAAATTATAATTTATAAGTGGCATTAGATGATTTTTATAAACCGAGAATCGAGTTTATATTAATAATTTTAAATTTAATATATTAAAATTTTAATAT

>EOG5B2WPB_DNA_query

aatcctcctcctagtgaaggtgtggttgttgtagaagttccaagattaaaagttggagtgctagtagtaagtgttccaaatgtgaaacctccagttgtttttggtagtccaaaacccatgtttgtagttgttgaaggttgggaaaatgtaaaacctgtaaaaaatttttaaatgacatgatttaagcttaaaaaaatttaaatgggctaatacatcaaaaaagtaaaattaagtaaaaattaaaaactgatttttattattgaataatgaattaataaataaaacaaaacttaataggagacaaattgtcatttagttaaaggaacaatatcaaaaaaatgcataaattaagatttcccaaaagacaaatcaaacttaaaactaaaatataattttgtgtagtatatagtatagtaataccagttgatgtagtggtagaaactgtagtaatacttgaagttacagatggattaactccagttccaaaaactaagtttgaagtagtgggtatagcagatcctaggtttgcatttggcattgtagatgaagcagaggtgacatttgctgaaggaaacgtaaatccagtattagatgtttgaggagtagaagatgcagttgttccaaacccagaaacatttgtagatccaaaggttccaaaggtagtcaaagatgtatttactgatggaaccccaaatgtaaaaccagttgaggtcacagaagtagttactggtggtttaactccaaaactggtattgaaagcaggagctccaggttgagttcccattaagtttgaaactcctgcagttccaaatgatgttgccgtgtttgtagtagcagcattggtaacagtactagaaaatccggtagaggcttgctccggtggtcttcaagcatttcaaaaattttaatgagatatatacaaaatttataaattattattaacaaatgtatagtacacaaaacgctttttcaaaattttaaatactcacttagaaccgaataaatttgttcccgatgaattaaaactaaaatttccaaacccagacatttgattgattaaaactta--------------------------------------------------------------------------------------------------------------------------------------------------nnnnnnnnnnnnnnnnnnnnnnnnnnnnnnnnnnnnnnnnnnnnnnnnnnnnnnnnnnnntatatttttagttgatatagtatttttttcgtcattattattaatcttttatatttttaaaatggattttcaaaatagagctggaggaaaaactggtggcggaggggtagcaacttggtcagaatctaatagagatcgaagagaacggttaagacaattggctttagaaacaattgatattaacaaagatccttattttatgaaaaatcacttaggttcttatgaatgtaaactgtgtttaactttacacaataatgaaggctcttatttggctcatactcaaggaaaaaaacatcaagctaatttggctagaagagctgctaaagatgctaaggatgcacctcaacaacctgcgcctgaaaagccaagggtagatgttaaaaaatttgtaaaaattggtaggccaggttacagagtcaccaagcaaagagatcctgattctggacaacaaagtttattatttcaggttcgtaatgatgttgaatataatacatatattattcatcttgaattcatttaaaaattacttaaattctctgtttaaaattaaaaaaaattaattaacattttgatttatgaaataaaaaatttttttttcccatttttaaaaatttagttatgattaatgtgtatttttttttttacagatagactacccagaaatagttgaaggtgttttaccacgccacagatttatgtctgcttatgaacagagagttgaaccccctgacaggaaatggcaatatttgttgtttgcagctgagccttacgaaacaatttcttttaaagtaattttgtttttaattaaaaatctgtatttattaggttattttttattttaaatttagtttaagtaatttttcataatgaatctacgtttggtgctttgcaacttagttttttaaattataatttgattgcttttttgctataatatgatgttaatttacagaaaaaaaactgttacaccttattaaaattgttattaaatttttttcaggtacctagtagagaagttgataaatcagatacaaaattttggactcactggaacaaagataccaagcagttttttttacaattttcattcaaaatagaacccaagcaacctcctaaaccaccaacaattggtaaccctcctcctccattaatgaaaaatcaaattttgccccctaactttgttcctccccctcccatgcctcctagaagtatggttcctcctcctccaagatcaataatgccctcagtagctcctccttctatgtttaatttaataccaccccctcctccaatagtcagtaccacatcaagtaatttaccaataccaccacctcctccttcacctctcctgagaaattaaactaactttaagttagtttttttaatttcatttgtatctacaatttaaagaaatgaaaatttcctaccaaaattttgctttatctattatctttacaattttatatttaatgcattaatttaatgtttaatgtaaattttgtgaaatcccattttttaaaattgataattttaaaaaataattaaaaataaatttaatttgctgtaaaataataatttttaaatacattctcacaaaaataaaatggacaagaaatttaatcaacattttaatgaacatgtgataattctgcattatgtgtaaaacagtgaatagtttttttctaaatatttaatagattatgataaaaaaaatagtattttttttctttttgcacacattaaatagcacttaatagtataggacaaattaaagacatttttaaataattaaaaaatttatttgatagaagaaatacaattatgcttggagaatacaggctcccccaacagctttaattttgtcttctgctctcttagaaaagaactttgccttgacaatgactggttgttttggtaatctacccttgccaagaactttgtaatagccctacgtaaaaaattacaatttattattatttagtattagcctagctttttaatcaattttttaaaatattacttacagctctaacaacatctataactggagcttttttttctttactattagcacatcttaatctagtgtctgcacttactagagtccaaattttatctaaattaatagctttgcaccatctagtatttctgttaagatgataatttctcattcccaactaaaaaattacaatttgatttattacaagaaatcaaaaaattaaaaattacttgcttgaattattacaagtttttcacctttccaaaataaccagggtggtatttgtcaaagttaattctgtggtggtgcatacctcctgcattacctctaccaccaggatgtttacggtgtttaccaatacgcccgtgtccatgtgaaacatgacctcttaatttacgagtcttcttcttattggcagcctattaataattattaaaagtattaatataatttttaaaccaaaaacattaataaaaaaaccaatggcttatgtttaaaatctaacatttttaattacag-------------------------------------------------------------------------------------------------------------------------------------------------------------------------------------------------------------------------------------------------------------------------------------------------------------

>EOG5B2WPM_Protein_query

---------------------------------------------------------------------------------------------------------------------------------------------------------------------------------------------------------------------------------------------------------------------------------------------------------------------------------------------------------------------------------------------------------------------------------AATAAGTAATTAAGAAAGGCGGTGAAATTTTCATTTTATTAATTTTGAATTTAAATTAAAACTTTTAATTCATTTTTCAAAGATTTTTACAAATATATTGTCAAAGTGTAAAATACTGAAAGTTTAAAATTAGGTATTATGTATCAGTGCTCCTAAAAAATGCATAAATTTTAACTTTTTAAAACAGTGAAAATTCGTAAGAGTGCATGGTGCATTCTAATTTTTTCTAATATTCATTTAGAGAAAATAAATTTTTTTTTTTTAATTGATTCTTAATTAAAAAT-AAAAAAAAAAAAACTTTAAAATAATTTATAATTATAAAATGAAAATTTAATATTTTAATTTGTTTCTTTAATTTGTAATAATTACTTTTAAGTTGTGCCAACTTGAGAACAAATAATTTAATTTTGGCAATAGTGTAATTCAATTTAAAATTTGACATACAAACACTCAACATTTTTCTATGAGATTCAATATAAAGTGATTTCATTTTATTAAATCTTAAAAACGCTTTTATAAAATTCAATATGGTTAACTTAATATTGGACATCGGGGCATTTCCCAGCAGAATTACTTTACCAGAAAATTTTATTTGTTCGGAACATGAGTTGAGACAAGCAGTTTTGAAACATTCCAAGAAAAATTATGCAAGTATATTTATTTTTTTATTAAAGCCAACAAGAACATTACTTAATTATAATATTCTAAAAAAATCAATATATTACTTTCAGGGTGCCACATTTGATTTTTATTTATTACGAAATGGAAAAATATTTTGTGAAAACACATATCAAGAATGTGACATACATGTGATACCCAGATTATGTGGGGGAAAGGGTGGATTTGGTTCCATGTTAAGAGCTATCGGAGCACAAATAGAAAAAACTACAAATAGGGAAGCTTGTCGAGATTTAAGTGGCAGAAGATTAAGAGATATAAATGAAGAAAAAAGATTAAAAAAATGGATAGCAGAGCAAGCGGAAAGAGAACAAGAAGCTGCTTTGAAAAAAAAGAAAAAATTGGAAAAATTAACAGAAATCACCAAACATGAATTTACAGATTCAGATTACACAAAAGCCAGACTTGAAATGCCAGAAAAAATTCATGAATCTCTTGAAGAAGGTTTAAAAGCAGCAGCAGCAGCAACAACAACAGAAGTTGAGGAAGAAAAAGAAAAAGAAACAGAAGCATGCACATCAAAAGAAATCAAACCTAAAGCGAAGAAAAAAAAACCTATCAAAGGAAATCTATGGTATTTTTTTTTTTTAAAAGAAATTCATCATTTTTAAATAAGCATAATTAAATTCAATCATACAAAAATAAAAATAAATTAATTTTTCTAATTTTAGGGTTGACGATATGGAATCATTAGAAGATGAATGTGATACATCCGAAAGCGAAAGTGATGACAAAGTAGTACCTAAGAAAAAGTTAAAAATATCTTGATAATATTTTTTTTTTTAAAATAAATAAACTAATTGTAAATACATATATAAAACTTCAAAATAAATTTTTTACATACATAAATTAAAATCATCTTTTTTTTATTATTATTAATTTTTTTAAAAACGTTAAATAATGACGTCATTTGGTTTTAGTCAAGCCTTTCGATATTACATCGGCATATCAAAAATTTAAAAAAAAATAATAATAATCTAATTGTAACAATTACGTTGGTTCATTTTCAAAAATAAATATAAATAAAAGGAATAATAATCATTAATAATAAATAAATAAATAAATAAATAAATTTTTTATCCCAACAAAAAAATATATATATATATTCCAATACAATATTTAATCAAATAATTACATTAAAATTAAAAGACAAAAAAAAGAAAAAAAA------------------------------------------------------------------------------------------------------------------------------------------------------------

>EOG5B2WPM_DNA_query

gcctgtcagtccgaactaatacacactaaaaaatcacttcaagaagtttaattttaaataaattttaacaaccacaacgttcgtatttcatattatctacaaactgacgaatagttataaacatacaaatatcagccatgtttgattgtttaaataatttcaatacaacgctctacggggttcacagttctaaatgaaggtctctccgcgataaaaaatgttttttattatttgaaatatacataatttaaaattttttttaaaaagctttgctaaataataaaatactaaaaattatttctgtaataaaaaaagaacaaaaaaattgcactaaaaataaataataaatgtataaatttagcgtaggaatatattttttatgacgtcattgtcatcagccattattcttgctttgacaataagtaattaagaaaggcggtgaaattttcattttattaattttgaatttaaattaaaacttttaattcatttttc-aagatttttacaaatatattgtcaaagtgtaaaatactgaaagtttaaaattaggtattatgtatcagtgctcctaaaaaatgcataaattttaactttttaaaacagtgaaaattcgtaagagtgcatggtgcattctaattttttctaatattcatttagagaaaataaattttttttttttaattgattcttaattaaaaataaaaaaaaaaaaaactttaaaataatttataattataaaatgaaaatttaatattttaatttgtttctttaatttgtaataattacttttaagttgtgccaacttgagaacaaataatttaattttggcaatagtgtaattcaatttaaaatttgacatacaaacactcaacatttttctatgagattcaatataaagtgatttcattttattaaatcttaaaaacgcttttataaaattcaatatggttagcttaatattggacatcggggcatttcccagcagaattactttaccagaaaattttatttgttcggaacatgagttgagacaagcagttttgaaacattccaagaaaaattatgcaagtatatttatttttttattaaagccaacaagaacattacttaattataatattctaaaaaaatcaatatattactttcagggtgccacatttgatttttatttattacgaaatggaaaaatattttgtgaaaacacatatcaagaatgtgacatacatgtgatacccagattatgtgggggaaagggtggatttggttccatgttaagagctatcggagcacaaatagaaaaaactacaaatagggaagcttgtcgagatttaagtggcagaagattaagagatataaatgaagaaaaaagattaaaaaaatggatagcagagcaagcggaaagagaacaagaagctgctttgaaaaaaaagaaaaaattggaaaaattaacagaaatcaccaaacatgaatttacagattcagattacacaaaagccagacttgaaatgccagaaaaaattcatgaatctcttgaagaaggtttaaaagcagcagcagcagcaacaacaacagaagttgaggaagaaaaagaaaaagaaacagaagcatgcacatcaaaagaaatcaaacctaaagcgaagaaaaaaaaacctatcaaaggaaatctatggtattttttttttttaaaagaaattcatcatttttaaataagcataattaaattcaatcatacaaaaataaaaataaattaatttttctaattttagggttgacgatatggaatcattagaagatgaatgtgatacatccgaaagcgaaagtgatgacaaagtagtacctaagaaaaagttaaaaatatcttgataatatttttttttttaaaataaataaactaattgtaaatacatatataaaacttcaaaataaattttttacatacataaattaaaatcatcttttttttattattattaatttttttaaaaacgttaaataatgacgtcatttggttttagtcaagcctttcgatattacatcggcatatcaaaaatttaaaaaaaaataataataatctaattgtaacaattacgttggttcattttcaaaaataaatataaataaaaggaataataatcattaataataaataaataaataaataaataaattttttatcccaacaaaaaaatatatatatatattccaatacaatatttaatcaaataattacattaaaattaaaagacaaaaaaaagaaaaaaaaaannnnnnnnnnnnnnnnnnnnnnnnnntctttcatcctttatatttgtgtatatactaaaatttgtattaaaaaaataacaaaattaatttcttatttattaataatatgtgaacaatcggatttaacttttattttttttaaaaaatgtttatc
