# Supplementary material for: aTRAM - automated target restricted assembly method: a fast method for assembling loci across divergent taxa from next-generation sequencing data
Source: BMC Bioinformatics. 2015 Mar 25;16(1):98. doi: 10.1186/s12859-015-0515-2 (PMC4380108; doi:10.1186/s12859-015-0515-2)
Supplement: Additional file 3: — Fasta files of 3 genes (CO1, EF1a and one unknown nuclear locus), from lice in the genus Degeeriella. Fasta files include aTRAM contigs as well as Sanger sequences. [file 12859_2015_515_MOESM3_ESM.docx]

**Additional File 3:**

**Allen et al., 2015 aTRAM: automated Target Restricted Assembly Method**

Aligned sequences assembled from aTRAM with sequences from Sanger sequencing, from two mitochondrial genes and one nuclear gene.

>aTRAM_Dgruf.Faber_CO1

TGAGGTGTACATTCTTATTCTTCCGGGATTTGGTTTAATTTCTCACATAGTTATAGAAGAAAGAGGTAAGAACCAAGTGTTTGGTAGATTAGGGATGATCTACGCCATGGTGTCTATTGGCATTTTAGGATTTATTGTGTGGGCACACCACATGTTTACTGTGGGGATAGATGTTGATAGTCGGGCATATTTTACTAGTGCAACAATGGTAATTGCAGTTCCTACCGGTATTAAGGTTTTTAGGTGGTTAGCTACCATGTTCGGGTCATCTAATTTAGGCTCGTCAAGATCTCTTTGGGCGCTGGGCTTTATTTTTCTGTTTACTGT

>SANGER_Dgruf.Faber.6.4.2012.1_CO1

TGAGGTGTACATTCTTATTCTTCCGGGATTTGGTTTAATTTCTCACATAGTTATAGAAGAAAGAGGTAAGAACCAAGTGTTTGGTAGATTAGGGATGATCTACGCCATGGTGTCTATTGGCATTTTAGGATTTATTGTGTGGGCACACCACATGTTTACTGTGGGGATAGATGTTGATAGTCGGGCATATTTTACTAGTGCAACAATGGTAATTGCAGTTCCTACCGGTATTAAGGTTTTTAGGTGGTTAGCTACCATGTTCGGGTCATCTAATTTAGGCTCGTCAAGATCTCTTTGGGCGCTGGGCTTTATTTTTCTGTTTACTGT

>SANGER_Dgruf.Faber.6.4.2012.1_EF1a

TGAAGAACGTCGCCGTGAAAGAATTGCGCAGAGGTTACGTCGCCGGCGACTCAAAGAACAATCCTCCGAAAGCGGCAGCCGATTTCCTTGCTCAGGTTATCGTCTTGAATCACCCAGGTCAAATTTCGAGCGGATATACTCCCGTGTTGGATTGCCACACGGCCCACATAGCCTGCAAGTTTGCTGAAATCAAGGAAAAATGTGACCGTCGTACTGGAAAGACTACGGAAGAGAACCCGAAGAGCATCAAGTCGGGAGACGCAGCCATCGTGAACTTGGTCCCCACGAAACCCATGTGCGTAGAATCTTTCTCCGAATTCCCGCCTCTTGGACGTTTCGCCGTCCGT

>aTRAM_Dgruf.Faber_EF1a

TGAAGAACGTCGCCGTGAAAGAATTGCGCAGAGGTTACGTCGCCGGCGACTCAAAGAACAATCCTCCGAAAGCGGCAGCCGATTTCCTTGCTCAGGTTATCGTCTTGAATCACCCAGGTCAAATTTCGAGCGGATATACTCCCGTGTTGGATTGCCACACGGCCCACATAGCCTGCAAGTTTGCTGAAATCAAGGAAAAATGTGACCGTCGTACTGGAAAGACTACGGAAGAGAACCCGAAGAGCATCAAGTCGGGAGACGCAGCCATCGTGAACTTGGTCCCCACGAAACCCATGTGCGTAGAATCTTTCTCCGAATTCCCGCCTCTTGGACGTTTCGCCGTCCGT

>SANGER_Dgruf.Faber.6.4.2012.1_Br50

AACCCCTGGAGGAAAATGTGAATAGGTTGCAGGTAGATGGACTGGAAGCTAGAACCATAACTGAAGCCATATCAATTCTAAGCACAAAGGATGAACCAGAAGACAGGCATCCAGAGAAACGAATGAAGGTGGCTTATGCAGCCTTTGAAGCTGCCAATTTACCNAGAATCAAAGCTGAAAATCCTACATTGAGGTTGTCTCAGTGGAAGCAAATTTTAAATAAAGACTGGATGAGATCTCC

>aTRAM_Dgruf.Faber_Br50

AACCCCTGGAGGAAAATGTGAATAGGTTGCAGGTAGATGGACTGGAAGCTAGAACCATAACTGAAGCCATATCAATTCTAAGCACAAAGGATGAACCAGAAGACAGGCATCCAGAGAAACGAATGAAGGTGGCTTATGCAGCCTTTGAAGCTGCCAATTTACCAAGAATCAAAGCTGAAAATCCTACATTGAGGTTGTCTCAGTGGAAGCAAATTTTAAATAAAGACTGGATGAGGTCACC
